# Bias magnification in ecologic studies: a methodological investigation

**DOI:** 10.1186/1476-069X-6-17

**Published:** 2007-07-05

**Authors:** Thomas F Webster

**Affiliations:** 1 Dept. of Environmental Health, Boston University School of Public Health, 715 Albany Street, Boston, MA 02118, USA

## Abstract

**Background:**

As ecologic studies are often inexpensive to conduct, consideration of the magnitude and direction of ecologic biases may be useful in both study design and sensitivity analysis of results. This paper examines three types of ecologic bias: confounding by group, effect measure modification by group, and non-differential exposure misclassification.

**Methods:**

Bias of the risk difference on the individual and ecologic levels are compared using two-by-two tables, simple equations, and risk diagrams. Risk diagrams provide a convenient way to simultaneously display information from both levels.

**Results:**

Confounding by group and effect measure modification by group act in the same direction on the individual and group levels, but have larger impact on the latter. The reduction in exposure variance caused by aggregation magnifies the individual level bias due to ignoring groups. For some studies, the magnification factor can be calculated from the ecologic data alone. Small magnification factors indicate little bias beyond that occurring at the individual level. Aggregation is also responsible for the different impacts of non-differential exposure misclassification on individual and ecologic studies.

**Conclusion:**

The analytical tools developed here are useful in analyzing ecologic bias. The concept of bias magnification may be helpful in designing ecologic studies and performing sensitivity analysis of their results.

## Background

Epidemiology is the study of health and disease in populations, but the standard for an observational study remains the individual level design, where we have information about outcome, exposure and covariates for each study subject [1]. This remains an ideal, although some designs mix group-level and individual-level variables in ways meant to enhance validity [2-5]. In practice, in the absence of better information, we often substitute an aggregate (group summary) value of some variable for each study subject. The extreme case is when aggregate values of exposure and outcome are used for every study variable. This is often called an ecologic study.

Resort to ecologic designs usually stems from the practical consideration that summary information is more easily obtained and more often available than individual-level data. Sometimes summary data are all that are available, and then, only in its crudest form, for example, that a certain percentage of a group of subjects is exposed (a group summary of an exposure variable) and a certain percentage of the same group has a specific health outcome (a group summary of an outcome variable). In this case we have lost information about whether those with the outcome are the same as those who are exposed. Despite this information loss, it is tempting and plausible to say that we still have some useful information on risks of exposure.

Epidemiologists know that using ecologic designs (group level variables only) to make inferences about individual risks (individual level variables) can be seriously biased [e.g., [6,7]], but exactly how and when this bias occurs is often mysterious. In discussions of individual-level studies it is not enough to say a result might be confounded; one should consider the amount and direction of confounding. Given the potential value that ecologic studies have for obtaining information not otherwise readily available, it would seem useful to approach these studies in the same way, i.e., not dismiss them at the outset but instead try to describe the magnitude and direction of potential biases.

Here I apply this idea to ecologic studies, using individual-level studies as a reference. In particular, I will discuss the direction and extent of bias in ecologic studies compared with studies of individuals. This paper is meant to reveal underlying mechanisms with a simple model so practicing epidemiologists can begin to visualize what is happening when aggregate data are used. Among the many types of bias possible in ecologic studies [7], I will examine three of the most important: confounding by group, effect measure modification by group, and non-differential exposure misclassification.

## Methods

### Use of two-by-two tables

Theoretical problems are often best approached by starting simply and adding complications later. I focus here on closed cohorts with binary exposures and outcomes, using the risk difference as an effect measure. This approach allows us to see the ecologic inference problem at work using simple tools.

Individual outcome and exposure data are readily summarized by the *interior cells *of a two-by-two table, i.e., the joint distribution of exposure and outcome (Table 1). From these data we easily compute the risks of the exposed and unexposed as well as the risk difference.

**Table 1:** Individual vs. ecologic data

	Individual (interior cells)			Ecologic (margins)		
		
	exposed	unexposed	sum	exposed	unexposed	sum
cases	16	12	28	?	?	28
noncases	24	48	72	?	?	72
total	40	60	100	40	60	100
risks	0.4	0.2		*X*	0.40	
RD	0.2			*Y*	0.28	
				*n*	100	

Individual-level information on exposure and outcome are shown by the interior of a two-by-two table; they are summarized by the risks in the unexposed and exposed and the risk difference (RD). Ecologic studies possess only the margins of the table, summarized by the average exposure *X*, average risk *Y *and group size *n*.

The ecologic data are also visible on the *margins *of the table [8]. They provide the average exposure and average risk for the whole group but not the exposed and unexposed subjects *within *the group.

### Risk diagrams and equations

**Figure 1:**
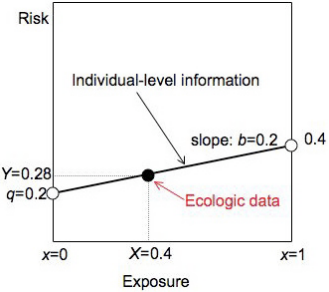
**Risk diagram illustrating Table 1**. We summarize individual-level information for a group with a solid black line, ecologic data with a solid black dot. The line connects the risk in the unexposed (*q *= 0.2 at *x *= 0) with the risk in the exposed (0.4 at *x *= 1) and has slope equal to the risk difference *b*. The ecologic data are the average exposure *X *and average risk *Y *for the group.

We can depict the information in a two-by-two table using a *risk diagram*, a graphical device adapted from earlier work [9-11]. Figure 1 presents a risk diagram for the example of Table 1. Risk is plotted on the vertical axis, exposure on the horizontal. For binary exposures, we plot the risk in the unexposed (0.2) at *x *= 0 and the risk in the exposed (0.4) at *x *= 1. The line connecting these points has a slope equal to the risk difference: (0.4-0.2)/(1-0) = 0.2. This line summarizes essential individual-level information in a two-by-two table: the risk difference, the risks in the exposed and the risks in the unexposed. We can represent the ecologic data for the table – the average exposure (*X *= 0.4) and the average risk (*Y *= 0.28) – by a large black dot. Thus risk diagrams show our knowledge about both levels, simultaneously: individual-level information is summarized by the line, ecologic data by the large dot.

For binary exposures, individual-level data only occur at exposures of zero and one, but it is convenient to think in terms of a continuous exposure. The simplest relationship would be a linear equation:

*r*_*j *_= *q *+ *bx*_*j*_

where *r*_*j *_is the risk as a function of exposure *x *and *j *is an index for subjects. The intercept *q *is the risk in the unexposed, also called the background risk. The slope *b *is the risk difference. We call these equations (linear) risk functions. They describe risk as a function of exposure on the individual level.

The expected value of the binary outcome for individual *y*_*j*_, considered as a probability, is equal to the person's risk, so that

*y*_*j *_= *q *+ *bx*_*j *_+ *e*_*j*_

where *e*_*j *_is an error term. One can also think of the risks as proportions and the *e*_*j *_as residuals (see appendix 1 for additional discussion of the model). Ordinary least squares regression of the individual-level data (*x*_*j*_, *y*_*j*_) in a two-by-two table can then be used to obtain the intercept *q *and risk difference *b*. We will see it is a useful tool for estimating ecologic bias. As discussed in appendix 2, this approach can be readily extended to rates, continuous outcomes (e.g., birth weights) and continuous exposures.

Since ecologic analyses only give us a single black dot for each two-by-two table, a collection of two-by-two tables is typically used. The idea is to extract information by examining how the outcome marginals vary as the exposure marginals change (e.g., how cancer rates change as the proportion of the population exposed to contaminated water changes across cities). This means we will usually be concerned with multiple tables, with each table describing a different group. We index the groups by the letter *i*:

*y*_*ij *_= *q*_*i *_+ *b*_*i*_*x*_*ij *_+ *e*_*ij*_

Since the background risk and risk difference may vary between groups, we must also add the index *i *to *q *and *b*.

Equations 1–3 describe individual-level models. In this paper, we will treat such models as a fixed reference for comparison with the results of ecologic inference. The fact that the *q*_*i *_and *b*_*i *_may differ between groups will prove critically important.

### Linearity and aggregation

If the risk function is linear, as in Figure 1, then the dot describing the ecologic data must lie on the line describing the individual-level information. For binary exposures, this occurs because the dot represents a weighted average of the exposed and unexposed. More generally, this fact is a consequence of the aggregation theorem [7]. Mathematically, this means that if the risk function is linear, the group-level equations produced by aggregating individual-level equations will have the same form and the same parameters (appendix 1). For example, averaging equation 3 within each group yields

*Y*_*i *_= *q*_*i *_+ *b*_*i*_*X*_*i*_

where *X*_*i *_and *Y*_*i *_are, respectively, the average exposure and average risk in group *i *(the aggregate error or residual term can typically be ignored). Following Susser [12], capital letters *X *and *Y *refer to group-level variables, lower-case *x *and *y *refer to individual-level variables.

**Figure 2:**
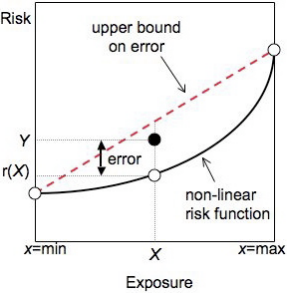
**Nonlinearity causes error during aggregation**. The ecologic data point (*X, Y*) will generally not fall on the risk function when the latter is non-linear as shown here. The amount of error depends on the curvature of the risk function and the exposure distribution, but is bounded above by the line connecting the risks at the minimum and maximum exposures. This error can lead to pure specification bias.

When the risk function is not linear, the equation describing aggregated data will generally not have the same form, and the ecologic data point will not lie on the risk function. Even if there are no other sources of bias, nonlinearity can thus cause trouble for ecologic studies – a problem called pure specification bias [7,9]. For example, suppose that exposure is a continuous variable and that the subjects in a group have the exposures denoted by the three open circles in Figure 2. The aggregate data, shown by the large dot, must then lie above the risk function (and below the line connecting the risks at the minimum and maximum exposures [11]). The average risk *Y *in the group is larger than the risk at the average exposure *r*(*X*) when the risk function is concave up. The difference between *Y *and *r*(*X*) depends on the shape of the risk function and the exposure distribution within groups [7,9]. Log-linear models introduce bias terms that can be approximated using within-group variances (appendix 2).

We continue our exposition using linear risk functions, showing how confounding and effect modification between groups and exposure misclassification increase bias when variables are aggregated.

### Loss of information and ecologic inference

**Figure 3:**
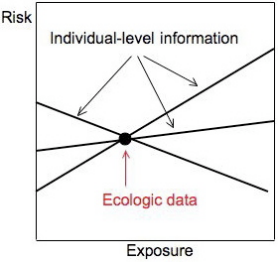
**Loss of information is a fundamental problem of ecologic studies**. Many sets of individual-level information (lines, interiors of two-by-two tables) generate the same ecologic data (dot, table margins). Only some possible lines are shown.

The traditional goal of ecologic inference is to draw conclusions about individuals based on group-level data, or equivalently: to deduce the interior of a two-by-two table from its margins, to obtain the line in a risk diagram from the dot, or to estimate the risk difference from the ecologic data (the average risks *Y*_*i *_and average exposures *X*_*i*_). This goal runs into the fundamental problem that ecologic studies suffer from a loss of information [10]. In terms of two-by-two tables, many tables with very different interior cell contents can have the same margins. In terms of risk diagrams, many lines can go through the same ecologic data point (Figure 3).

A single dot is insufficient to determine a line. What if there were two or more dots, i.e., several two-by-two tables? Could we then recover the individual level information? The answer is "Yes," but only by making some very strong assumptions. If the assumptions are violated, large biases can occur.

## Results

Epidemiologists often use regression of data from a number of groups for ecologic inference, regressing the average risk *Y*_*i *_in each group against the average exposure *X*_*i *_in each group. This approach is sometimes called ecologic or Goodman regression [10,13] (For a more formal treatment of ecologic regression and other methods, as well as ecologic bias, see [14]). We will use weighted least squares, weighting each group by its population *n*_*i*_. Unweighted regression of ecologic data can cause an additional source of bias relative to individual-level analysis (appendix 3).

Ecologic regression *can *produce unbiased results. One way is to assume the individual-level model has the same background risk (intercept *q*) and risk difference (slope *b*) in every group:

*y*_*ij *_= *q *+ *bx*_*ij *_+ *e*_*ij*_

Aggregating yields the equation

*Y*_*i *_= *q *+ *bX*_*i*_

Ecologic regression then yields the correct estimate of the risk difference *b*. Assuming *q*_*i *_= *q *and *b*_*i *_= *b *is not the only way to achieve unbiased results, but it is the easiest to understand. In terms of risk diagrams, the lines describing the individual-level information in every group coincide. Since the dots representing the ecologic data all lie on this line, the ecologic regression reproduces the individual-level result.

But if *q *and *b *differ between the groups, things can go wrong. This difference corresponds to confounding and effect measure modification between groups. In an important paper, Greenland and Morgenstern [15] described these sources of ecologic bias. We use the analytic framework described above, the risk diagram and the elegant work of Palmquist [16] to show how the magnitude and direction of the ecologic bias from these sources affects biases present at the individual-level.

### Confounding by group

Suppose two groups have the same risk difference *b *but different background risks, *q*_0 _≠ *q*_1_:

*y*_0*j *_= *q*_0 _+ *bx*_0*j *_+ *e*_0*j*_

*y*_1*j *_= *q*_1 _+ *bx*_1*j *_+ *e*_1*j*_

**Figure 4:**
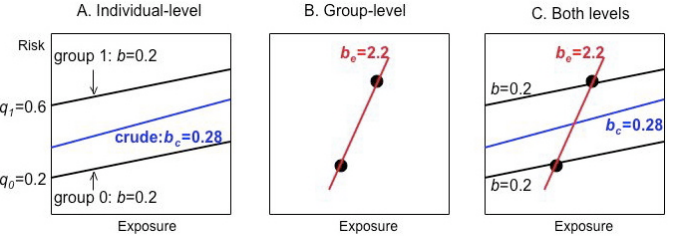
**Confounding by group, illustrating Table 2**. **A) **Individual level: The solid black lines describing the individual-level information in the two groups are parallel (same risk differences *b*) but have different intercepts (different background risks *q*_0 _≠ *q*_1_). The crude estimate of the risk difference *b*_*c *_is confounded (blue line). **B) **Group level: The ecologic estimate of the risk difference *b*_*e *_is the slope of the red line through the two ecologic data points. Massive confounding has occurred, but we can't tell this from the ecologic data alone. **C) **Comparison of results on the two levels: The ecologic estimate of the risk difference *b*_*e *_is much more biased than the crude individual-level estimate *b*_*c*_. Both biases are in the same direction.

Figure 4A is a risk diagram illustrating the example of Table 2. The lines describing the individual-level information for the two groups are parallel because they have the same risk difference, but have different intercepts because the background risks are not equal. As the exposure distributions of the two groups also differ (since *X*_0 _≠ *X*_1_), ignoring groups causes confounding on the individual level.

**Table 2:** Confounding by group

	Group 0			Group 1			Crude		
			
	expose	unexposed	sum	expose	unexpose	sum	expose	unexpose	sum
case	16	12	28	48	24	72	64	36	100
noncase	24	48	72	12	16	28	36	64	100
total	40	60	100	60	40	100	100	100	200
risk	0.40	0.20		0.80	0.60		0.64	0.36	
RD	0.20			0.20			0.28		
*X*_*i*_	0.40			0.60					
*Y*_*i*_	0.28			0.72					
*n*_*i*_	100			100					

Since background risks and exposure distributions differ between the two groups, the crude individual-level estimate of the risk difference is confounded. The ecologic estimate of the risk difference, (*Y*_1_-*Y*_0_)/(*X*_1_-*X*_0_) = 2.2, is much more confounded. RD = risk difference; *X*_*i *_and *Y*_*i *_are the average exposure and average risk in group *i; n*_*i *_= size of group *i*.

The line describing the crude individual-level information in Table 2 (the table obtained from combining both groups) has a somewhat higher slope, i.e., confounding by group biased the crude risk difference upward (we use the word bias in an epidemiologic sense, the difference between an estimate and the correct value, *b*). If we know the individual-level data, including the variable describing group, we can prevent confounding by controlling for group, either by stratifying or adding group as a covariate in a regression.

Figure 4B shows the ecologic data (*X*_*i*_, *Y*_*i*_) for the two groups and the result of an ecologic regression. We know something has gone wrong, since a risk difference cannot exceed one, but we cannot determine the source of the problem from ecologic data alone. Unlike the individual case, we cannot control for group by stratifying or including an indicator variable in the regression: the ecologic data provide insufficient information for using these techniques (e.g., with only two ecologic data points, we cannot add a covariate to the ecologic regression).

Figure 4C plots the ecologic and crude individual-level results on the same risk diagram. The biases are in the same direction, but much larger for the ecologic study. Indeed, the ecologic bias is 25 times larger than the crude individual-level bias:

be−bbc−b=2.2−0.20.28−0.2=25
 MathType@MTEF@5@5@+=feaafiart1ev1aaatCvAUfKttLearuWrP9MDH5MBPbIqV92AaeXatLxBI9gBaebbnrfifHhDYfgasaacH8akY=wiFfYdH8Gipec8Eeeu0xXdbba9frFj0=OqFfea0dXdd9vqai=hGuQ8kuc9pgc9s8qqaq=dirpe0xb9q8qiLsFr0=vr0=vr0dc8meaabaqaciaacaGaaeqabaqabeGadaaakeaadaWcaaqaaiabdkgaInaaBaaaleaacqWGLbqzaeqaaOGaeyOeI0IaemOyaigabaGaemOyai2aaSbaaSqaaiabdogaJbqabaGccqGHsislcqWGIbGyaaGaeyypa0ZaaSaaaeaacqaIYaGmcqGGUaGlcqaIYaGmcqGHsislcqaIWaamcqGGUaGlcqaIYaGmaeaacqaIWaamcqGGUaGlcqaIYaGmcqaI4aaocqGHsislcqaIWaamcqGGUaGlcqaIYaGmaaGaeyypa0JaeGOmaiJaeGynaudaaa@48CA@

*b*_*e *_and *b*_*c *_are, respectively, the ecologic and crude individual-level estimates of the risk difference *b*. In appendix 3 we show that the relative amounts of bias due to confounding by group equals the exposure variance on the individual level divided by the exposure variance on the group level:

be−bbc−b=var⁡[xij]var⁡B[Xi]=25
 MathType@MTEF@5@5@+=feaafiart1ev1aaatCvAUfKttLearuWrP9MDH5MBPbIqV92AaeXatLxBI9gBaebbnrfifHhDYfgasaacH8akY=wiFfYdH8Gipec8Eeeu0xXdbba9frFj0=OqFfea0dXdd9vqai=hGuQ8kuc9pgc9s8qqaq=dirpe0xb9q8qiLsFr0=vr0=vr0dc8meaabaqaciaacaGaaeqabaqabeGadaaakeaadaWcaaqaaiabdkgaInaaBaaaleaacqWGLbqzaeqaaOGaeyOeI0IaemOyaigabaGaemOyai2aaSbaaSqaaiabdogaJbqabaGccqGHsislcqWGIbGyaaGaeyypa0ZaaSaaaeaacyGG2bGDcqGGHbqycqGGYbGCcqGGBbWwcqWG4baEdaWgaaWcbaGaemyAaKMaemOAaOgabeaakiabc2faDbqaaiGbcAha2jabcggaHjabckhaYnaaBaaaleaacqqGcbGqaeqaaOGaei4waSLaemiwaG1aaSbaaSqaaiabdMgaPbqabaGccqGGDbqxaaGaeyypa0JaeGOmaiJaeGynaudaaa@50A8@

var[*x*_*ij*_] is the total exposure variance on the individual level and var_B_[*X*_*i*_] is the exposure variance on the ecologic level (the between-group variance) weighted using the population of each group.

### Bias magnification of confounding by group

We can rewrite equation 9 as

(*b*_*e *_- *b*) = (*b*_*c *_- *b*) *M*

The amount of ecologic confounding by group (*b*_*e *_- *b*) equals the amount of individual-level confounding by group (*b*_*c *_- *b*) times a *magnification factor M*:

M=var⁡[xij]var⁡B[Xi]
 MathType@MTEF@5@5@+=feaafiart1ev1aaatCvAUfKttLearuWrP9MDH5MBPbIqV92AaeXatLxBI9gBaebbnrfifHhDYfgasaacH8akY=wiFfYdH8Gipec8Eeeu0xXdbba9frFj0=OqFfea0dXdd9vqai=hGuQ8kuc9pgc9s8qqaq=dirpe0xb9q8qiLsFr0=vr0=vr0dc8meaabaqaciaacaGaaeqabaqabeGadaaakeaacqWGnbqtcqGH9aqpdaWcaaqaaiGbcAha2jabcggaHjabckhaYjabcUfaBjabdIha4naaBaaaleaacqWGPbqAcqWGQbGAaeqaaOGaeiyxa0fabaGagiODayNaeiyyaeMaeiOCai3aaSbaaSqaaiabbkeacbqabaGccqGGBbWwcqWGybawdaWgaaWcbaGaemyAaKgabeaakiabc2faDbaaaaa@44AF@

See Palmquist [16] for a closely related result.

Applying equations 10–11 to our example (Table 2 and Figure 4) shows that a moderate amount of confounding on the individual level (0.28-0.2 = 0.08) is magnified 25 times, producing a huge amount of confounding (2.2-0.2 = 2) on the ecologic level:

M=var⁡[xij]var⁡B[Xi]=0.250.01=25(2.2−0.2)=(0.28−0.2)252=(0.08)25
 MathType@MTEF@5@5@+=feaafiart1ev1aaatCvAUfKttLearuWrP9MDH5MBPbIqV92AaeXatLxBI9gBaebbnrfifHhDYfgasaacH8akY=wiFfYdH8Gipec8Eeeu0xXdbba9frFj0=OqFfea0dXdd9vqai=hGuQ8kuc9pgc9s8qqaq=dirpe0xb9q8qiLsFr0=vr0=vr0dc8meaabaqaciaacaGaaeqabaqabeGadaaakeaafaqabeWabaaabaGaemyta0Kaeyypa0ZaaSaaaeaacyGG2bGDcqGGHbqycqGGYbGCcqGGBbWwcqWG4baEdaWgaaWcbaGaemyAaKMaemOAaOgabeaakiabc2faDbqaaiGbcAha2jabcggaHjabckhaYnaaBaaaleaacqqGcbGqaeqaaOGaei4waSLaemiwaG1aaSbaaSqaaiabdMgaPbqabaGccqGGDbqxaaGaeyypa0ZaaSaaaeaacqaIWaamcqGGUaGlcqaIYaGmcqaI1aqnaeaacqaIWaamcqGGUaGlcqaIWaamcqaIXaqmaaGaeyypa0JaeGOmaiJaeGynaudabaGaeiikaGIaeGOmaiJaeiOla4IaeGOmaiJaeyOeI0IaeGimaaJaeiOla4IaeGOmaiJaeiykaKIaeyypa0JaeiikaGIaeGimaaJaeiOla4IaeGOmaiJaeGioaGJaeyOeI0IaeGimaaJaeiOla4IaeGOmaiJaeiykaKIaeGOmaiJaeGynaudabaGaeGOmaiJaeyypa0JaeiikaGIaeGimaaJaeiOla4IaeGimaaJaeGioaGJaeiykaKIaeGOmaiJaeGynaudaaaaa@6DC2@

Several conclusions follow immediately from equation 10. If there is no confounding by group on the individual level (*b*_*c *_- *b *= 0), there is no confounding by group on the ecologic level: *b*_*e *_- *b *= 0. Furthermore, since *M *is always positive, both biases are in the same direction. Suppose, as in the example, that we use the mean exposure in each group as the ecologic measure of exposure. *M *is then always *at least *one, i.e., the amount of confounding by group on the ecologic level equals or exceeds the amount on the individual level (appendix 4). (Note that the derivation of (10) assumes that var_B_[*X*_*i*_] is non-zero. When this assumption is violated, as occurs when the *X*_*i *_are equal in all groups, there is no confounding by group on the individual level. However, ecologic regression is uninformative since division by zero makes *b*_*e *_and *M *undefined).

Equation (10) tells us that the relative amount of confounding by group on the ecologic and individual level stems from the reduction of exposure variance caused by aggregation. The information loss from discarding within-group exposure variance magnifies the bias already present on the crude individual level. However, if exposure within groups is homogeneous, i.e., everyone within a group has the same exposure, *M *equals one and the amount of confounding by group is equal on the ecologic and individual levels. This formalizes, for one source of bias, the simple idea that ecologic studies with homogeneous exposures are really just individual-level studies.

**Figure 5:**
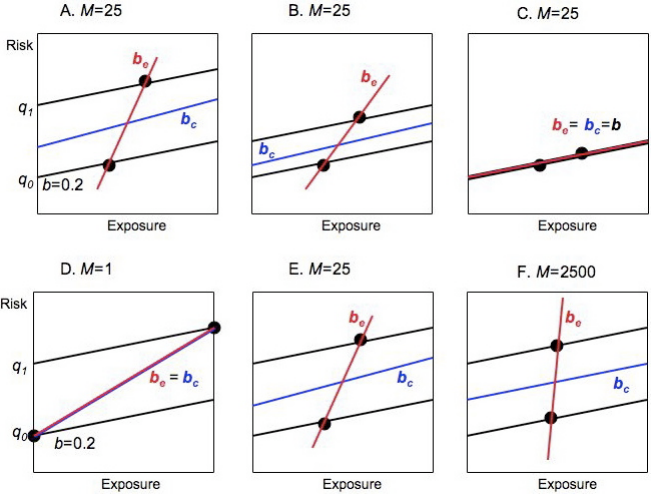
**Confounding by group on the individual and group level**. **A, B, C) **Suppose average exposures are the same, but the difference between the background risks (*q*_*i*_) decreases. Confounding by group decreases on both the individual and group levels with constant proportionality factor *M*. **D, E, F) **Suppose background risks (*q*_*i*_) are the same, but the difference between the average exposures decreases. Confounding by group decreases on the individual level, but increases on the ecologic level because of the large increase in *M*.

Changes in the background risks (*q*_*i*_) and average exposures (*X*_*i*_) have different implications for confounding on the individual and group levels. As shown in Figure 5, keeping average exposures the same but making the background risks more similar decreases confounding by group on both the individual and group levels. However, keeping background risks the same but making average exposures more similar (decreasing var_B_[*X*_*i*_]) decreases confounding by group on the individual level but increases it on the ecologic level: the decrease in *b*_*c *_- *b *is outweighed by the increase in *M*. Thus even a little confounding on the individual level can produce a lot on the ecologic level.

### Effect modification of the risk difference by group

Suppose two groups have the same background risk (*q*) but different risk differences (*b*_0 _≠ *b*_1_):

*y*_0*j *_= *q *+ *b*_0_*x*_0*j *_+ *e*_0*j*_

*y*_1*j *_= *q *+ *b*_1_*x*_1*j *_+ *e*_1*j*_

**Figure 6:**
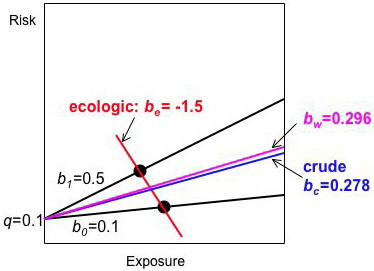
**Effect modification of the risk difference by group, illustrating Table 3**. The solid black lines describing the individual-level information for the two groups have the same intercept (background risk *q*) but different slopes (risk differences *b*_0 _≠ *b*_1_). The crude estimate of the risk difference *b*_*c *_(blue line) lies between these two extremes. Relative to *b*_*w*_, the ecologic estimate of the risk difference *b*_*e *_(red line) is far more biased than the crude individual-level estimate *b*_*c*_. Both biases are in the same direction. *b*_*w *_(purple line) is the weighted average of the risk differences used in the bias magnification equation.

The lines describing the two groups in Figure 6 have the same intercept but different slopes. As Figure 6 and Table 3 illustrate, the crude individual-level risk difference *b*_*c *_lies between the *b*_*i *_of the two groups, as it must for binary exposures (see appendix 5). In contrast, the ecologic estimate of the risk difference *b*_*e *_is wildly biased, not even having the same sign.

**Table 3:** Effect modification of the risk difference by group

	Group 0			Group 1			Crude		
			
	expose	unexpose	sum	expose	unexpose	sum	expose	unexpose	sum
case	20	10	30	48	12	60	68	22	90
noncase	80	90	170	32	108	140	112	198	310
total	100	100	200	80	120	200	180	220	400
risk	0.2	0.1		0.6	0.1		0.378	0.1	
RD	0.1			0.5			0.278		
*X*_*i*_	0.5			0.4					
*Y*_*i*_	0.15			0.3					
*n*_*i*_	200			200					

The background risks in each group are the same but the risk differences vary. The crude individual-level risk difference lies between the two extremes, as it must when exposure is binary. The ecologic estimate of the risk difference, (*Y*_1_-*Y*_0_)/(*X*_1_-*X*_0_)= -1.5, is extremely biased. RD = risk difference; *X*_*i *_and *Y*_*i *_are the average exposure and average risk in group *i; n*_*i *_= size of group *i*.

We can use equation 10 with one small change to describe the implications of effect modification of the risk difference by group. Since *b *is no longer constant, we use *b*_*w*_, a weighted average of the risk differences *b*_*i *_in the groups with weights depending on the within-group exposure variances (*b*_*w *_is also obtained by regressing the individual-level data while adjusting for group; see appendix 3):

(*b*_*e *_- *b*_*w*_) = (*b*_*c *_- *b*_*w*_) *M*

In the example, *b*_*w *_is approximately 0.296 (appendix 6). Allowing for rounding error, applying equation 14 yields

M=var⁡[xij]var⁡B[Xi]=0.24750.0025=99(−1.5−0.296)=(0.278−0.296)99−1.796=(−0.018)99
 MathType@MTEF@5@5@+=feaafiart1ev1aaatCvAUfKttLearuWrP9MDH5MBPbIqV92AaeXatLxBI9gBaebbnrfifHhDYfgasaacH8akY=wiFfYdH8Gipec8Eeeu0xXdbba9frFj0=OqFfea0dXdd9vqai=hGuQ8kuc9pgc9s8qqaq=dirpe0xb9q8qiLsFr0=vr0=vr0dc8meaabaqaciaacaGaaeqabaqabeGadaaakeaafaqabeWabaaabaGaemyta0Kaeyypa0ZaaSaaaeaacyGG2bGDcqGGHbqycqGGYbGCcqGGBbWwcqWG4baEdaWgaaWcbaGaemyAaKMaemOAaOgabeaakiabc2faDbqaaiGbcAha2jabcggaHjabckhaYnaaBaaaleaacqqGcbGqaeqaaOGaei4waSLaemiwaG1aaSbaaSqaaiabdMgaPbqabaGccqGGDbqxaaGaeyypa0ZaaSaaaeaacqaIWaamcqGGUaGlcqaIYaGmcqaI0aancqaI3aWncqaI1aqnaeaacqaIWaamcqGGUaGlcqaIWaamcqaIWaamcqaIYaGmcqaI1aqnaaGaeyypa0JaeGyoaKJaeGyoaKdabaGaeiikaGIaeyOeI0IaeGymaeJaeiOla4IaeGynauJaeyOeI0IaeGimaaJaeiOla4IaeGOmaiJaeGyoaKJaeGOnayJaeiykaKIaeyypa0JaeiikaGIaeGimaaJaeiOla4IaeGOmaiJaeG4naCJaeGioaGJaeyOeI0IaeGimaaJaeiOla4IaeGOmaiJaeGyoaKJaeGOnayJaeiykaKIaeGyoaKJaeGyoaKdabaGaeyOeI0IaeGymaeJaeiOla4IaeG4naCJaeGyoaKJaeGOnayJaeyypa0JaeiikaGIaeyOeI0IaeGimaaJaeiOla4IaeGimaaJaeGymaeJaeGioaGJaeiykaKIaeGyoaKJaeGyoaKdaaaaa@7E61@

As Figure 6 illustrates, the tiny discrepancy between the crude risk difference *b*_*c *_and the weighted average *b*_*w *_is multiplied by a large magnification factor of 99, producing a large bias on the ecologic level. The difference between *b*_*c *_and *b*_*w *_is not usually considered a bias on the individual level. For individual-level studies, some epidemiologists might report the *b*_*i *_if they consider the variation between groups important; others might ignore it. While *b*_*w *_is not a commonly used data summary of the *b*_*i*_, it helps us understand a source of ecologic bias when there is effect modification of the risk difference by group.

### Magnification factor

**Figure 7:**
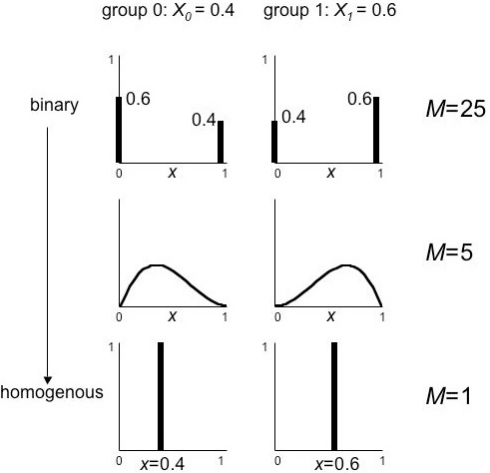
**Effect on *M *of different within-group exposure distributions**. The magnification factor *M *decreases when the within-group exposure variance is reduced, keeping the between-group variance constant (the *X*_*i *_do not change between rows).

The magnification factor is the linchpin of the mechanism. As Figure 5D–F illustrates, if the exposure distribution changes so that *M *increases, the amount of ecologic bias can increase dramatically. Another example may provide a better feel for the magnification factor. In Figure 7, we keep the average exposures (*X*_*i*_) the same in all three cases; the between-group variance (var_B_[*X*_*i*_]) thus remains constant. Changing the within-group exposure distribution from binary to homogeneous reduces the within-group variance (var[*x*_*ij*_]). As a result, *M *decreases from 25 to 1.

### Bias magnification

The *bias magnification equation *(equation 14), governs bias from both sources – confounding by group and effect modification of the risk difference by group – in an additive fashion, i.e., it can be applied to both sources of bias separately or together [11]. Application of the bias magnification equation to these sources of ecologic bias brings together two lines of research. Greenland and Morgenstern showed that both confounding by group and effect measure modification by group could cause ecologic bias [15]. The bias magnification equation can be derived by partitioning covariance and variance within and between groups (appendix 3). This approach has a distinguished history, only some of it mentioned here. Robinson's landmark 1950 paper [17] discussed such partitions in terms of correlation coefficients. Duncan *et al*. discussed regression coefficients [18]. Piantadosi *et al*. derived the bias magnification equation, but they did not emphasize the magnification factor or discuss the role of effect measure modification by group [19]. Palmquist derived a generalized form of a closely related equation using matrix methods [16]. Palmquist's insightful work, discussed by King [10], stresses the role of the inflation factor – the magnification factor minus one – and its effect on individual-level bias (appendix 3). Palmquist and King do not discuss the individual-level bias (which they call the specification shift) in terms of confounding and effect measure modification, in part because these authors are social scientists. King considers *b*_*c *_as his parameter of interest, biased *by *grouping. In our context, which is epidemiology, the crude risk difference *b*_*c *_is considered biased by *ignoring *groups.

### Non-differential misclassification of binary exposure

Here, non-differential exposure misclassification (NDEM) means that the proportion of people misclassified by exposure does not depend on disease status. Sensitivity *s *refers to the proportion of exposed people classified as exposed; specificity *t *means the proportion of non-exposed people classified as non-exposed (More general definitions may regard *s *and *t *as probabilities). NDEM causes bias towards the null in individual-level studies, but away from the null in ecologic studies [20]. This difference has been called one of the most significant problems of ecologic studies [6], so we conclude this exposition with an explanation of the mechanism in this simple case.

We compute misclassified individual and group level data for a two by two table as shown in Table 4. Since the misclassification is non-differential, the average exposure is affected but the average risk *Y*_*i *_is not changed. This is the key to the effect. The average exposure *U*_*i *_in the misclassified table is given by

**Table 4:** Non-differential exposure misclassification in a 2 × 2 table

	Correct			Misclassified		
		
	expose	unexpose	sum	expose	unexpose	sum
Case	*a*_*i*_	*b*_*i*_	*a*_*i*_+*b*_*i*_	*sa*_*i *_+ (1-*t*)*b*_*i*_	(1-*s*)*a*_*i*_+*tb*_*i*_	*a*_*i*_+*b*_*i*_
noncase	*c*_*i*_	*d*_*i*_	*c*_*i*_+*d*_*i*_	*sc*_*i *_+ (1-*t*)*d*_*i*_	(1-*s*)*c*_*i*_+*td*_*i*_	*c*_*i*_+*d*_*i*_
Total	*a*_*i*_+*c*_*i*_	*b*_*i*_+*d*_*i*_	*n*_*i*_	*s*(*a*_*i*_+*c*_*i*_) + (1-*t*)(*b*_*i*_+*d*_*i*_)	(1-*s*)(*a*_*i*_+*c*_*i*_) + *t*(*b*_*i*_+*d*_*i*_)	*n*_*i*_
*X*_*i*_	(*a*_*i*_+*c*_*i*_)*/n*_*i*_			*s*(*a*_*i*_+*c*_*i*_)*/n*_*i *_+ (1-*t*)(*b*_*i*_+*d*_*i*_)*/n*_*i*_		
*Y*_*i*_	(*a*_*i*_+*b*_*i*_)*/n*_*i*_			(*a*_*i*_+*b*_*i*_)*/n*_*i*_		

Assume that the proportion of people misclassified does not depend on disease status. Sensitivity *s *is the fraction of exposed people classified as exposed; specificity *t *is the fraction of unexposed people classified as unexposed. On the group level, the average risk remains the same but the average exposure changes. *X*_*i *_and *Y*_*i *_are the average exposure and average risk in the group; *n*_*i *_= size of the group.

*U*_*i *_= *λX*_*i *_+ (1 - *t*)

*λ *= *s *+ *t *- 1

where *λ *is *Youden's index *[1] (for details of this section, see appendix 7). Since sensitivity and specificity must be between zero and one, *λ *must be between -1 and 1. Sensitivity and specificity are typically greater than 0.5 (i.e., better than random), so we assume *λ *is between 0 and 1.

With this background, it is easy to show that ecologic studies are biased away from the null when sensitivity and specificity are the same in every group [20]. The ecologic estimate of the risk difference for the misclassified data (*b*_*e*_') equals

be′=beλ
 MathType@MTEF@5@5@+=feaafiart1ev1aaatCvAUfKttLearuWrP9MDH5MBPbIqV92AaeXatLxBI9gBamXvP5wqSXMqHnxAJn0BKvguHDwzZbqegyvzYrwyUfgarqqtubsr4rNCHbGeaGqiA8vkIkVAFgIELiFeLkFeLk=iY=Hhbbf9v8qqaqFr0xc9pk0xbba9q8WqFfeaY=biLkVcLq=JHqVepeea0=as0db9vqpepesP0xe9Fve9Fve9GapdbaqaaeGacaGaaiaabeqaamqadiabaaGcbaGaemOyai2aa0baaSqaaiabdwgaLbqaaOGamai4gkdiIcaacqGH9aqpdaWcaaqaaiabdkgaInaaBaaaleaacqWGLbqzaeqaaaGcbaacciGae83UdWgaaaaa@4851@

When *λ *is between 0 and 1, *b*_*e*_' is farther away from the null than the true ecologic estimate and has the same sign.

**Figure 8:**
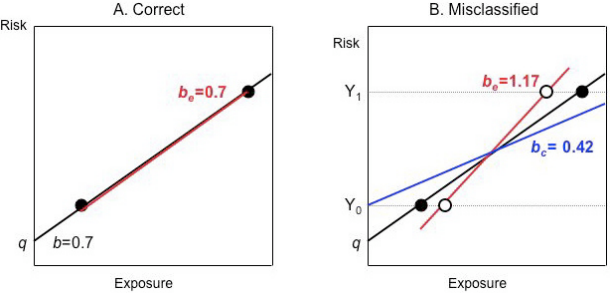
**Non-differential exposure misclassification (NDEM), illustrating Table 5**. **A**. If there are no other sources of bias, the ecologic- and individual-level analyses of the correct data are the same. **B**. Suppose the dichotomous exposure data are misclassified with the same sensitivity and specificity in each group. Then the individual-level result (blue) is biased toward the null and the ecologic result (red) is biased away from the null. The average risks (*Y*_*i*_) in each group are unchanged but the average exposures move closer together. This causes the resulting ecologic regression line to have higher slope.

Figure 8 shows how NDEM works in this simple model. Suppose we have an ecologic study of two groups with no other sources of bias (Table 5). The true ecologic data lie on top of the line describing the underlying individual level information. NDEM has no effect on the average risks *Y*_*i*_, so the height of the dots describing the ecologic data stays the same. NDEM causes the misclassified average exposures *U*_*i *_to move closer together and towards the center compared with the true average exposures *X*_*i*_. Consequently, the regression line through the ecologic data is steeper, i.e., biased away from the null. The different effects of NDEM on the individual and group levels is ultimately due to the loss of information caused by aggregation. While average risks are unaffected, aggregation brings the average exposures closer together, i.e., the same risks are produced by a narrower range of exposures, resulting in a steeper slope.

**Table 5:** Effect of non-differential exposure misclassification on individual and ecologic studies

	Group 0			Group 1			Crude		
			
*Correct*									
	expose	unexpose	sum	expose	unexpose	sum	expose	unexpose	sum

cases	160	80	240	720	10	730	880	90	970
noncases	40	720	760	180	90	270	220	810	1030
total	200	800	1000	900	100	1000	1100	900	2000
risk	0.8	0.1		0.8	0.1		0.8	0.1	
RD	0.7			0.7			0.7		
*X*_*i*_	0.20			0.90					
*Y*_*i*_	0.24			0.73					

*Misclassify*									

	expose	unexpose	sum	expose	unexpose	sum	expose	unexpose	sum

cases	144	96	240	578	152	730	722	248	970
noncases	176	584	760	162	108	270	338	692	1030
total	320	680	1000	740	260	1000	1060	940	2000
risk	0.45	0.14		0.78	0.58		0.68	0.26	
RD	0.31			0.20			0.42		
*X*_*i*_	0.32			0.74					
*Y*_*i*_	0.24			0.73					

The data are misclassified assuming the same sensitivity and specificity in each group, both equal to 0.8. Since there are no other sources of bias in this example, the crude individual and ecologic estimates of the RD are identical (0.7) for the correct data. For the misclassified data, the crude individual level estimate of the RD (0.42) is biased toward the null while the ecologic estimate (1.17) is biased away from the null. RD = risk difference; *X*_*i *_and *Y*_*i *_are the average exposure and average risk in group *i*.

It is important to note that not all forms of exposure measurement error will bias ecologic studies away from the null. We examined a particular error model above: NDEM of binary exposure at the individual level with sensitivities and specificities not changing between groups. Other error models lead to other results [11]. For example, application of the classical error model to a continuous exposure variable biases results toward the null in ecologic studies.

## Discussion

Roughly speaking, the bias magnification equation says that ecologic bias equals individual-level bias magnified. More precisely, the reduction in exposure variance caused by aggregation (loss of information) magnifies the individual-level bias due to confounding by group and/or effect modification of the risk difference by group. Other things equal, the magnification factor is maximized if exposure within groups is dichotomous [11]. Thus textbook examples, which typically use two-by-two tables, tend to overstate the amount of bias magnification occurring in many real studies.

Bias magnification provides a useful tool for theoretical considerations of ecologic bias. Does it have any practical use? When designing ecologic studies, one should try to minimize *M *by increasing between-group differences in exposure while making within-group exposure as homogeneous as possible; see also [7]. Bias magnification also suggests an approach to sensitivity analysis of ecologic bias from confounding by group and/or effect modification of the risk difference by group. Assume an individual-level model as a reference. The ecologic bias has two components: the amount of bias caused by ignoring group on the individual-level (*b*_*c *_- *b*_*w*_), and the magnification of this bias caused by aggregation (*M*). When analyzing ecologic data, we will not know the size of the bias on the individual level, but we can make various assumptions. For example, we may be able to make educated guesses about the direction and possible amount of confounding by group on the individual level. We may then be able to estimate the magnification factor. For binary exposures, we can compute *M *from the ecologic data alone (appendix 8). In other situations we may be able to estimate *M *from samples of the study population or from routinely collected environmental data. For example, we might use air pollution measurements and spatial statistics to estimate the variation in exposure within cities, comparing it to variation in average air pollution between cities. If *M *is small, we gain confidence that the ecologic bias isn't too different from the bias on the individual level. See [21] for another approach to sensitivity analysis of ecologic bias.

Ecologic studies using exposure variables of the type "fraction exposed" may be particularly problematic. Such ecologic exposure variables are the aggregated form of binary exposures on the individual level. Other things equal, use of such variables tends to maximize the magnification factor (increasing any bias present due to confounding by group or effect measure modification by group), increase bias in non-linear models, and bias results away from the null when non-differential exposure misclassification occurs. Studies with small variation between average exposures (as in Figure 5F) are particularly worrisome.

The approach discussed here examines three important sources of ecologic bias: confounding by group, effect modification of the risk difference by group, and non-differential exposure misclassification. It does not take into account other potential problems, e.g., confounding within groups or effect measure modification within groups (However, confounding within groups alone does not cause ecologic bias when the relationship between exposure and outcome is linear; see appendix 9. The plausibility of the linearity assumption must be carefully considered).

In this paper we have employed a simple, abstract approach based on several assumptions: availability of the same types of information on both individual and group levels, use of average group exposure as the ecologic exposure measure, linear models, weighted least squares regression, estimation of the risk difference. Our purpose was to display, as simply as possible, the underlying mechanisms causing the magnification of individual bias upon aggregation into groups. The loss of information about within-group exposure variance is seen to be the culprit.

The ideas here can be extended, with some modifications of results, to examine additional issues: inclusion of covariates, confounding and effect measure modification within groups, group-level exposure measures other than means, and partially ecologic studies [4,11]. This paper has focused on the risk difference, but the results can be readily applied to the rate difference and studies with continuous outcomes (appendix 3). Generalization to the more commonly used relative risks and rate ratios is underway (see also [21]).

In addition to theoretical investigations like this one, we also need to know more about the amount of ecologic bias encountered in practice [7,22-24]. By helping us focus on how ecologic studies go astray, we hope to move toward the goal of domesticating ecologic bias: treating it as another source of epidemiologic bias that needs to be analyzed and quantified [22].

## Mathematical appendix

### 1. Linear risk functions and aggregation

Assume risk to individuals is a linear function of exposure *x*

*r*_*ij *_= *q*_*i *_+ *b*_*i*_*x*_*ij*_

We index groups with *i *and subjects with *j*, and allow the background risk *q*_*i *_and risk difference *b*_*i *_to vary between groups (Alternatively, one can think in terms of an unmeasured group-level covariate *Z*_*i*_: *q*_*i *_= *q *+ *γZ*_*i*_, *b*_*i *_= *b *+ *ηZ*_*i*_). Conceiving of risks as probabilities of developing disease, the expected value of the binary outcome for individual *y*_*ij *_is equal to their risk, so that

*y*_*ij *_= *q*_*i *_+ *b*_*i*_*x*_*ij *_+ *e*_*ij*_

where *e*_*ij *_is an error term. Averaging within groups (of size *n*_*i*_) produces the aggregate equation

1ni∑j=1niyij=1ni∑j=1ni(qi+bixij+eij)=qi+bi(1ni∑j=1nixij)+1ni∑j=1nieij
 MathType@MTEF@5@5@+=feaafiart1ev1aaatCvAUfKttLearuWrP9MDH5MBPbIqV92AaeXatLxBI9gBaebbnrfifHhDYfgasaacH8akY=wiFfYdH8Gipec8Eeeu0xXdbba9frFj0=OqFfea0dXdd9vqai=hGuQ8kuc9pgc9s8qqaq=dirpe0xb9q8qiLsFr0=vr0=vr0dc8meaabaqaciaacaGaaeqabaqabeGadaaakeaadaWcaaqaaiabigdaXaqaaiabd6gaUnaaBaaaleaacqWGPbqAaeqaaaaakmaaqahabaGaemyEaK3aaSbaaSqaaiabdMgaPjabdQgaQbqabaaabaGaemOAaOMaeyypa0JaeGymaedabaGaemOBa42aaSbaaWqaaiabdMgaPbqabaaaniabggHiLdGccqGH9aqpdaWcaaqaaiabigdaXaqaaiabd6gaUnaaBaaaleaacqWGPbqAaeqaaaaakmaaqahabaGaeeikaGIaemyCae3aaSbaaSqaaiabdMgaPbqabaGccqGHRaWkcqWGIbGydaWgaaWcbaGaemyAaKgabeaakiabdIha4naaBaaaleaacqWGPbqAcqWGQbGAaeqaaOGaey4kaSIaemyzau2aaSbaaSqaaiabdMgaPjabdQgaQbqabaGccqqGPaqkaSqaaiabdQgaQjabg2da9iabigdaXaqaaiabd6gaUnaaBaaameaacqWGPbqAaeqaaaqdcqGHris5aOGaeyypa0JaemyCae3aaSbaaSqaaiabdMgaPbqabaGccqGHRaWkcqWGIbGydaWgaaWcbaGaemyAaKgabeaakmaabmaabaWaaSaaaeaacqaIXaqmaeaacqWGUbGBdaWgaaWcbaGaemyAaKgabeaaaaGcdaaeWbqaaiabdIha4naaBaaaleaacqWGPbqAcqWGQbGAaeqaaaqaaiabdQgaQjabg2da9iabigdaXaqaaiabd6gaUnaaBaaameaacqWGPbqAaeqaaaqdcqGHris5aaGccaGLOaGaayzkaaGaey4kaSYaaSaaaeaacqaIXaqmaeaacqWGUbGBdaWgaaWcbaGaemyAaKgabeaaaaGcdaaeWbqaaiabdwgaLnaaBaaaleaacqWGPbqAcqWGQbGAaeqaaaqaaiabdQgaQjabg2da9iabigdaXaqaaiabd6gaUnaaBaaameaacqWGPbqAaeqaaaqdcqGHris5aaaa@88A5@

*Y*_*i *_= *q*_*i *_+ *b*_*i*_*X*_*i *_+ *e*_*i*_

*X*_*i *_and *Y*_*i *_are the average exposure and outcome per group (See [14,21] for an in-depth discussion of statistical models in ecologic studies). The *e*_*ij *_and *e*_*i *_vanish under expectation.

Alternatively, we can think of the risks as proportions. Under the proportion model, the *e*_*ij *_and *e*_*i *_are residuals (*e*_*i *_then vanishes in equation A4 because the sum of residuals within groups equals zero). Both the probability and proportion interpretations can be applied in this paper: the proportion model may help in thinking about ecologic bias in particular data sets. For example, the individual-level model(s) assumed for an analysis need not be correct; instead it is a reference against which we measure ecologic bias.

We assumed a linear risk model (A2) at the individual level. Although very simple, it still yields insight into many aspects of ecologic bias (Non-linear functions, described below, add some additional features). Linear risk models can also be easily analyzed using ordinary least squares (OLS). The risk difference (*b*_*i*_) is the natural effect measure to use in this situation. For example, applying OLS to the individual-level data in a two-by-two table yields *q*_*i *_and *b*_*i *_(OLS picks the line that runs through the mean values of the outcomes at the two exposure levels). Although OLS is not commonly used for analyzing binary outcome data, it simplifies the understanding of ecologic bias. OLS, and other methods described here, are directly applicable to many studies of continuous outcomes.

While we chose to use a linear risk model, the relationship between the average outcome in a group (*Y*_*i*_) and the average exposure (*X*_*i*_) in two-by-two tables must be linear [10,11]; using the notation in Table 6, the average risk in the group is given by:

**Table 6:** Notation for a general two-by-two table

	exposed	unexposed	sum
cases	*p*_*i*_*m*_*i*1_	*q*_*i*_*m*_*i*0_	*p*_*i*_*m*_*i*1_+*q*_*i*_*m*_*i*0_
noncases	(1-*p*_*i*_)*m*_*i*1_	(1-*q*_*i*_)*m*_*i*0_	(1-*p*_*i*_)*m*_*i*1 _+ (1-*q*_*i*_)*m*_*i*0_
total	*m*_*i*1_	*m*_*i*0_	*n*_*i *_= *m*_*i*1_+*m*_*i*0_
risks	*p*_*i*_	*q*_*i*_	
RD	*p*_*i*_-*q*_*i*_		
*X*_*i*_			*m*_*i*1_*/n*_*i*_
*Y*_*i*_			(*p*_*i*_*m*_*i*1_+*q*_*i*_*m*_*i*0_)/*n*_*i*_

Let *p*_*i *_and *q*_*i *_be the risks of the exposed and unexposed in group *i*. Let *m*_*i*1 _and *m*_*i*0 _be the numbers of exposed and unexposed in group *i*. RD = risk difference; *X*_*i *_and *Y*_*i *_are the average exposure and average risk in group *i; n*_*i *_= size of group *i*.

Yi=pimi1+qimi0ni=piXi+qi(1−Xi)=qi+(pi−qi)Xi=qi+biXi
 MathType@MTEF@5@5@+=feaafiart1ev1aaatCvAUfKttLearuWrP9MDH5MBPbIqV92AaeXatLxBI9gBaebbnrfifHhDYfgasaacH8akY=wiFfYdH8Gipec8Eeeu0xXdbba9frFj0=OqFfea0dXdd9vqai=hGuQ8kuc9pgc9s8qqaq=dirpe0xb9q8qiLsFr0=vr0=vr0dc8meaabaqaciaacaGaaeqabaqabeGadaaakqaaeeqaaiabdMfaznaaBaaaleaacqWGPbqAaeqaaOGaeyypa0ZaaSaaaeaacqWGWbaCdaWgaaWcbaGaemyAaKgabeaakiabd2gaTnaaBaaaleaacqWGPbqAcqaIXaqmaeqaaOGaey4kaSIaemyCae3aaSbaaSqaaiabdMgaPbqabaGccqWGTbqBdaWgaaWcbaGaemyAaKMaeGimaadabeaaaOqaaiabd6gaUnaaBaaaleaacqWGPbqAaeqaaaaakiabg2da9iabdchaWnaaBaaaleaacqWGPbqAaeqaaOGaemiwaG1aaSbaaSqaaiabdMgaPbqabaGccqGHRaWkcqWGXbqCdaWgaaWcbaGaemyAaKgabeaakiabcIcaOiabigdaXiabgkHiTiabdIfaynaaBaaaleaacqWGPbqAaeqaaOGaeiykaKIaeyypa0JaemyCae3aaSbaaSqaaiabdMgaPbqabaGccqGHRaWkcqGGOaakcqWGWbaCdaWgaaWcbaGaemyAaKgabeaakiabgkHiTiabdghaXnaaBaaaleaacqWGPbqAaeqaaOGaeiykaKIaemiwaG1aaSbaaSqaaiabdMgaPbqabaaakeaacqGH9aqpcqWGXbqCdaWgaaWcbaGaemyAaKgabeaakiabgUcaRiabdkgaInaaBaaaleaacqWGPbqAaeqaaOGaemiwaG1aaSbaaSqaaiabdMgaPbqabaaaaaa@6DF2@

Equation A5 is identical to A4 (except for *e*_*i*_).

### 2. Extensions; non-linear risk functions and pure specification bias

Equations 1–3 (in the main text) and Figure 1 were constructed to describe two-by-two tables and risks, but the approach is readily extended to rates, continuous outcomes and continuous exposures. Instead of modeling risks in a closed cohort, we can also examine incident cases in person-time [11]. In this type of individual-level study, we would know the interior of the table (the number of exposed and unexposed cases); we could therefore compute the rates for the exposed and unexposed as well as the rate difference. In an ecologic study of this type, we would know only the marginal rate and the marginal exposure distribution. Rate diagrams are very similar to risk diagrams except that the *y *axis has no upper bound. For continuous outcomes *y*_*ij *_with normally distributed errors *e*_*ij*_, OLS is a conventional model of analysis; *b *is then the change in outcome per unit change in exposure. Instead of restricting *x*_*ij *_to zero or one as in a binary exposure, we can also let *x*_*ij *_be a continuous measure of exposure. For equations 1–3 (and Figure 1) to hold, the risk function would have to be linear but non-linear functions are also possible.

Instead of the linear risk function (A2), suppose we assume the following log-linear model:

*y*_*ij *_= exp[*q *+ *bx*_*ij*_] + *e*_*ij*_

Aggregating yields (ignoring error terms)

Yi=1ni∑j=1niexp⁡[q+bxij]
 MathType@MTEF@5@5@+=feaafiart1ev1aaatCvAUfKttLearuWrP9MDH5MBPbIqV92AaeXatLxBI9gBaebbnrfifHhDYfgasaacH8akY=wiFfYdH8Gipec8Eeeu0xXdbba9frFj0=OqFfea0dXdd9vqai=hGuQ8kuc9pgc9s8qqaq=dirpe0xb9q8qiLsFr0=vr0=vr0dc8meaabaqaciaacaGaaeqabaqabeGadaaakeaacqWGzbqwdaWgaaWcbaGaemyAaKgabeaakiabg2da9maalaaabaGaeGymaedabaGaemOBa42aaSbaaSqaaiabdMgaPbqabaaaaOWaaabCaeaacyGGLbqzcqGG4baEcqGGWbaCcqGGBbWwcqWGXbqCcqGHRaWkcqWGIbGycqWG4baEdaWgaaWcbaGaemyAaKMaemOAaOgabeaakiabc2faDbWcbaGaemOAaOMaeyypa0JaeGymaedabaGaemOBa42aaSbaaWqaaiabdMgaPbqabaaaniabggHiLdaaaa@4BAB@

Equation A7 is generally not equal to exp[*q*+*bX*_*i*_], i.e., for non-linear models the individual-level and aggregate models do not have the same functional form. This discrepancy is the source of pure specification bias [7,9]. If *x*_*ij *_is normally distributed within groups, then applying expectations and cumulants to (A6) yields:

Yi=exp⁡[q+bXi+b22σi2]
 MathType@MTEF@5@5@+=feaafiart1ev1aaatCvAUfKttLearuWrP9MDH5MBPbIqV92AaeXatLxBI9gBaebbnrfifHhDYfgasaacH8akY=wiFfYdH8Gipec8Eeeu0xXdbba9frFj0=OqFfea0dXdd9vqai=hGuQ8kuc9pgc9s8qqaq=dirpe0xb9q8qiLsFr0=vr0=vr0dc8meaabaqaciaacaGaaeqabaqabeGadaaakeaacqWGzbqwdaWgaaWcbaGaemyAaKgabeaakiabg2da9iGbcwgaLjabcIha4jabcchaWnaadmaabaGaemyCaeNaey4kaSIaemOyaiMaemiwaG1aaSbaaSqaaiabdMgaPbqabaGccqGHRaWkdaWcaaqaaiabdkgaInaaCaaaleqabaGaeGOmaidaaaGcbaGaeGOmaidaaGGaciab=n8aZnaaDaaaleaacqWGPbqAaeaacqaIYaGmaaaakiaawUfacaGLDbaaaaa@45B0@

where σi2
 MathType@MTEF@5@5@+=feaafiart1ev1aaatCvAUfKttLearuWrP9MDH5MBPbIqV92AaeXatLxBI9gBaebbnrfifHhDYfgasaacH8akY=wiFfYdH8Gipec8Eeeu0xXdbba9frFj0=OqFfea0dXdd9vqai=hGuQ8kuc9pgc9s8qqaq=dirpe0xb9q8qiLsFr0=vr0=vr0dc8meaabaqaciaacaGaaeqabaqabeGadaaakeaaiiGacqWFdpWCdaqhaaWcbaGaemyAaKgabaGaeGOmaidaaaaa@30F0@ is the exposure variance within group *i *[9]. More generally, we can approximate (A8) by applying Taylor series and aggregation to (A6) (see also [25-27]).

Assuming that counts of cases fit a Poisson model, one can fit ecologic data using (A8). One can, however, gain some insight into pure specification bias using an approximation. Linearly regressing *X*_*i *_against log[*Y*_*i*_] yields:

be≈b+b22cov⁡[Xi,σi2]var⁡[Xi]
 MathType@MTEF@5@5@+=feaafiart1ev1aaatCvAUfKttLearuWrP9MDH5MBPbIqV92AaeXatLxBI9gBaebbnrfifHhDYfgasaacH8akY=wiFfYdH8Gipec8Eeeu0xXdbba9frFj0=OqFfea0dXdd9vqai=hGuQ8kuc9pgc9s8qqaq=dirpe0xb9q8qiLsFr0=vr0=vr0dc8meaabaqaciaacaGaaeqabaqabeGadaaakeaacqWGIbGydaWgaaWcbaGaemyzaugabeaakiabgIKi7kabdkgaIjabgUcaRmaalaaabaGaemOyai2aaWbaaSqabeaacqaIYaGmaaaakeaacqaIYaGmaaWaaSaaaeaacyGGJbWycqGGVbWBcqGG2bGDcqGGBbWwcqWGybawdaWgaaWcbaGaemyAaKgabeaakiabcYcaSGGaciab=n8aZnaaDaaaleaacqWGPbqAaeaacqaIYaGmaaGccqGGDbqxaeaacyGG2bGDcqGGHbqycqGGYbGCcqGGBbWwcqWGybawdaWgaaWcbaGaemyAaKgabeaakiabc2faDbaaaaa@4F02@

The exponential of *b*_*e *_now estimates the relative risk. As shown by (A9), in the absence of other sources of ecologic bias, log-linear ecologic regression is subject to pure specification bias, approximated by the second term in (A9). For such models, the within-group exposure variances can often be expected to covary with average exposures. There is little or no bias if *b *is small (low curvature), the within group exposure variance (σi2
 MathType@MTEF@5@5@+=feaafiart1ev1aaatCvAUfKttLearuWrP9MDH5MBPbIqV92AaeXatLxBI9gBaebbnrfifHhDYfgasaacH8akY=wiFfYdH8Gipec8Eeeu0xXdbba9frFj0=OqFfea0dXdd9vqai=hGuQ8kuc9pgc9s8qqaq=dirpe0xb9q8qiLsFr0=vr0=vr0dc8meaabaqaciaacaGaaeqabaqabeGadaaakeaaiiGacqWFdpWCdaqhaaWcbaGaemyAaKgabaGaeGOmaidaaaaa@30F0@) does not depend on *X*_*i*_, or exposure is uniform within groups (σi2
 MathType@MTEF@5@5@+=feaafiart1ev1aaatCvAUfKttLearuWrP9MDH5MBPbIqV92AaeXatLxBI9gBaebbnrfifHhDYfgasaacH8akY=wiFfYdH8Gipec8Eeeu0xXdbba9frFj0=OqFfea0dXdd9vqai=hGuQ8kuc9pgc9s8qqaq=dirpe0xb9q8qiLsFr0=vr0=vr0dc8meaabaqaciaacaGaaeqabaqabeGadaaakeaaiiGacqWFdpWCdaqhaaWcbaGaemyAaKgabaGaeGOmaidaaaaa@30F0@ = 0), results consistent with those found by Richardson *et al*. [9] for the normal distribution case. Risk diagrams drawn using log-transformed risks turn exponential risk functions into straight lines; the line describing the upper bound of the error in Figure 2 becomes curved (bowing downward).

### 3. Bias magnification equation

Assume the individual-level model of equation A2. The crude individual-level and population-weighted ecologic estimates of the risk difference are, respectively:

bc=cov⁡[yij,xij]var⁡[xij]=CTVT
 MathType@MTEF@5@5@+=feaafiart1ev1aaatCvAUfKttLearuWrP9MDH5MBPbIqV92AaeXatLxBI9gBaebbnrfifHhDYfgasaacH8akY=wiFfYdH8Gipec8Eeeu0xXdbba9frFj0=OqFfea0dXdd9vqai=hGuQ8kuc9pgc9s8qqaq=dirpe0xb9q8qiLsFr0=vr0=vr0dc8meaabaqaciaacaGaaeqabaqabeGadaaakeaacqWGIbGydaWgaaWcbaGaem4yamgabeaakiabg2da9maalaaabaGagi4yamMaei4Ba8MaeiODayNaei4waSLaemyEaK3aaSbaaSqaaiabdMgaPjabdQgaQbqabaGccqGGSaalcqWG4baEdaWgaaWcbaGaemyAaKMaemOAaOgabeaakiabc2faDbqaaiGbcAha2jabcggaHjabckhaYjabcUfaBjabdIha4naaBaaaleaacqWGPbqAcqWGQbGAaeqaaOGaeiyxa0faaiabg2da9maalaaabaGaem4qam0aaSbaaSqaaiabdsfaubqabaaakeaacqWGwbGvdaWgaaWcbaGaemivaqfabeaaaaaaaa@521F@

be=cov⁡B[Yi,Xi]var⁡B[Xi]=CBVB
 MathType@MTEF@5@5@+=feaafiart1ev1aaatCvAUfKttLearuWrP9MDH5MBPbIqV92AaeXatLxBI9gBaebbnrfifHhDYfgasaacH8akY=wiFfYdH8Gipec8Eeeu0xXdbba9frFj0=OqFfea0dXdd9vqai=hGuQ8kuc9pgc9s8qqaq=dirpe0xb9q8qiLsFr0=vr0=vr0dc8meaabaqaciaacaGaaeqabaqabeGadaaakeaacqWGIbGydaWgaaWcbaGaemyzaugabeaakiabg2da9maalaaabaGagi4yamMaei4Ba8MaeiODay3aaSbaaSqaaiabbkeacbqabaGccqGGBbWwcqWGzbqwdaWgaaWcbaGaemyAaKgabeaakiabcYcaSiabdIfaynaaBaaaleaacqWGPbqAaeqaaOGaeiyxa0fabaGagiODayNaeiyyaeMaeiOCai3aaSbaaSqaaiabbkeacbqabaGccqGGBbWwcqWGybawdaWgaaWcbaGaemyAaKgabeaakiabc2faDbaacqGH9aqpdaWcaaqaaiabdoeadnaaBaaaleaacqWGcbGqaeqaaaGcbaGaemOvay1aaSbaaSqaaiabdkeacbqabaaaaaaa@4F86@

*V*_*T *_and *V*_*B *_are the total and between-group exposure variances (This derivation assumes *V*_*T *_and *V*_*B *_are non-zero). *C*_*T *_and *C*_*B *_are the total and between-group covariances of outcome and exposure. *C*_*B *_and *V*_*B *_are weighted by group size *n*_*i*_:

CB=cov⁡B[Xi,Yi]=1n∑i=0N−1niXiYi−(X¯)(Y¯)
 MathType@MTEF@5@5@+=feaafiart1ev1aaatCvAUfKttLearuWrP9MDH5MBPbIqV92AaeXatLxBI9gBaebbnrfifHhDYfgasaacH8akY=wiFfYdH8Gipec8Eeeu0xXdbba9frFj0=OqFfea0dXdd9vqai=hGuQ8kuc9pgc9s8qqaq=dirpe0xb9q8qiLsFr0=vr0=vr0dc8meaabaqaciaacaGaaeqabaqabeGadaaakeaacqWGdbWqdaWgaaWcbaGaemOqaieabeaakiabg2da9iGbcogaJjabc+gaVjabcAha2naaBaaaleaacqWGcbGqaeqaaOGaei4waSLaemiwaG1aaSbaaSqaaiabdMgaPbqabaGccqGGSaalcqWGzbqwdaWgaaWcbaGaemyAaKgabeaakiabc2faDjabg2da9maalaaabaGaeGymaedabaGaemOBa4gaamaaqahabaGaemOBa42aaSbaaSqaaiabdMgaPbqabaGccqWGybawdaWgaaWcbaGaemyAaKgabeaakiabdMfaznaaBaaaleaacqWGPbqAaeqaaOGaeyOeI0IaeiikaGIafmiwaGLbaebacqGGPaqkcqGGOaakcuWGzbqwgaqeaiabcMcaPaWcbaGaemyAaKMaeyypa0JaeGimaadabaGaemOta4KaeyOeI0IaeGymaedaniabggHiLdaaaa@59E6@

VB=var⁡B[Xi]=1n∑i=0N−1niXi2−(X¯)2
 MathType@MTEF@5@5@+=feaafiart1ev1aaatCvAUfKttLearuWrP9MDH5MBPbIqV92AaeXatLxBI9gBaebbnrfifHhDYfgasaacH8akY=wiFfYdH8Gipec8Eeeu0xXdbba9frFj0=OqFfea0dXdd9vqai=hGuQ8kuc9pgc9s8qqaq=dirpe0xb9q8qiLsFr0=vr0=vr0dc8meaabaqaciaacaGaaeqabaqabeGadaaakeaacqWGwbGvdaWgaaWcbaGaemOqaieabeaakiabg2da9iGbcAha2jabcggaHjabckhaYnaaBaaaleaacqWGcbGqaeqaaOGaei4waSLaemiwaG1aaSbaaSqaaiabdMgaPbqabaGccqGGDbqxcqGH9aqpdaWcaaqaaiabigdaXaqaaiabd6gaUbaadaaeWbqaaiabd6gaUnaaBaaaleaacqWGPbqAaeqaaOGaemiwaG1aa0baaSqaaiabdMgaPbqaaiabikdaYaaaaeaacqWGPbqAcqGH9aqpcqaIWaamaeaacqWGobGtcqGHsislcqaIXaqma0GaeyyeIuoakiabgkHiTiabcIcaOiqbdIfayzaaraGaeiykaKYaaWbaaSqabeaacqaIYaGmaaaaaa@5298@

where *N *is the number of groups, *n *is the total population, and X¯
 MathType@MTEF@5@5@+=feaafiart1ev1aaatCvAUfKttLearuWrP9MDH5MBPbIqV92AaeXatLxBI9gBaebbnrfifHhDYfgasaacH8akY=wiFfYdH8Gipec8Eeeu0xXdbba9frFj0=OqFfea0dXdd9vqai=hGuQ8kuc9pgc9s8qqaq=dirpe0xb9q8qiLsFr0=vr0=vr0dc8meaabaqaciaacaGaaeqabaqabeGadaaakeaacuWGybawgaqeaaaa@2DFD@ and Y¯
 MathType@MTEF@5@5@+=feaafiart1ev1aaatCvAUfKttLearuWrP9MDH5MBPbIqV92AaeXatLxBI9gBaebbnrfifHhDYfgasaacH8akY=wiFfYdH8Gipec8Eeeu0xXdbba9frFj0=OqFfea0dXdd9vqai=hGuQ8kuc9pgc9s8qqaq=dirpe0xb9q8qiLsFr0=vr0=vr0dc8meaabaqaciaacaGaaeqabaqabeGadaaakeaacuWGzbqwgaqeaaaa@2DFF@ are the overall means. Expand equation A10, partitioning the total covariance and variance into within-group and between-group pieces [18,19]:

bc=CTVT=CW+CBVT=CWVWVWVT+CBVBVBVT=bwVWVT+beVBVT
 MathType@MTEF@5@5@+=feaafiart1ev1aaatCvAUfKttLearuWrP9MDH5MBPbIqV92AaeXatLxBI9gBaebbnrfifHhDYfgasaacH8akY=wiFfYdH8Gipec8Eeeu0xXdbba9frFj0=OqFfea0dXdd9vqai=hGuQ8kuc9pgc9s8qqaq=dirpe0xb9q8qiLsFr0=vr0=vr0dc8meaabaqaciaacaGaaeqabaqabeGadaaakeaafaqadeWabaaabaGaemOyai2aaSbaaSqaaiabdogaJbqabaGccqGH9aqpdaWcaaqaaiabdoeadnaaBaaaleaacqWGubavaeqaaaGcbaGaemOvay1aaSbaaSqaaiabdsfaubqabaaaaOGaeyypa0ZaaSaaaeaacqWGdbWqdaWgaaWcbaGaem4vaCfabeaakiabgUcaRiabdoeadnaaBaaaleaacqWGcbGqaeqaaaGcbaGaemOvay1aaSbaaSqaaiabdsfaubqabaaaaaGcbaGaeyypa0ZaaSaaaeaacqWGdbWqdaWgaaWcbaGaem4vaCfabeaaaOqaaiabdAfawnaaBaaaleaacqWGxbWvaeqaaaaakmaalaaabaGaemOvay1aaSbaaSqaaiabdEfaxbqabaaakeaacqWGwbGvdaWgaaWcbaGaemivaqfabeaaaaGccqGHRaWkdaWcaaqaaiabdoeadnaaBaaaleaacqWGcbGqaeqaaaGcbaGaemOvay1aaSbaaSqaaiabdkeacbqabaaaaOWaaSaaaeaacqWGwbGvdaWgaaWcbaGaemOqaieabeaaaOqaaiabdAfawnaaBaaaleaacqWGubavaeqaaaaaaOqaaiabg2da9iabdkgaInaaBaaaleaacqWG3bWDaeqaaOWaaSaaaeaacqWGwbGvdaWgaaWcbaGaem4vaCfabeaaaOqaaiabdAfawnaaBaaaleaacqWGubavaeqaaaaakiabgUcaRiabdkgaInaaBaaaleaacqWGLbqzaeqaaOWaaSaaaeaacqWGwbGvdaWgaaWcbaGaemOqaieabeaaaOqaaiabdAfawnaaBaaaleaacqWGubavaeqaaaaaaaaaaa@679B@

*C*_*W *_and *V*_*W *_are the within-group covariance and variance. Equation A14 shows that the crude individual estimate of the risk difference *b*_*c *_is a weighted average of the within-group and ecologic estimates: *b*_*w *_≤ *b*_*c *_≤ *b*_*e *_since *V*_*W *_+ *V*_*B *_= *V*_*T *_and variances are nonnegative. *b*_*w*_, equal to *C*_*W*_/*V*_*W*_, is the within-group individual-level estimate of the risk difference. It is a weighted average of the *b*_*i *_with weights equal to *n*_*i *_var_i_(*x*_*ij*_), where var_i_(*x*_*ij*_) is the exposure variance within group *i*. *b*_*w *_is also the result of ordinary least squares regression of the individual-level data, adjusted for group using indicator variables [11,16]. After substituting *V*_*W *_= *V*_*T*_ - *V*_*B*_, a little algebra yields the bias magnification equation:

(be−bw)=(bc−bw)VTVB=(bc−bw)M
 MathType@MTEF@5@5@+=feaafiart1ev1aaatCvAUfKttLearuWrP9MDH5MBPbIqV92AaeXatLxBI9gBaebbnrfifHhDYfgasaacH8akY=wiFfYdH8Gipec8Eeeu0xXdbba9frFj0=OqFfea0dXdd9vqai=hGuQ8kuc9pgc9s8qqaq=dirpe0xb9q8qiLsFr0=vr0=vr0dc8meaabaqaciaacaGaaeqabaqabeGadaaakeaadaqadaqaaiabdkgaInaaBaaaleaacqWGLbqzaeqaaOGaeyOeI0IaemOyai2aaSbaaSqaaiabdEha3bqabaaakiaawIcacaGLPaaacqGH9aqpdaqadaqaaiabdkgaInaaBaaaleaacqWGJbWyaeqaaOGaeyOeI0IaemOyai2aaSbaaSqaaiabdEha3bqabaaakiaawIcacaGLPaaadaWcaaqaaiabdAfawnaaBaaaleaacqWGubavaeqaaaGcbaGaemOvay1aaSbaaSqaaiabdkeacbqabaaaaOGaeyypa0ZaaeWaaeaacqWGIbGydaWgaaWcbaGaem4yamgabeaakiabgkHiTiabdkgaInaaBaaaleaacqWG3bWDaeqaaaGccaGLOaGaayzkaaGaemyta0eaaa@4DC9@

**Figure 9:**
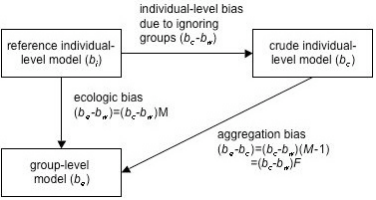
**Bias magnification and inflation**. The ecologic bias (*b*_*e *_- *b*_*w*_) equals the individual-level bias (*b*_*c *_- *b*_*w*_) – due to confounding by group and effect modification of the risk difference by group – multiplied by the magnification factor *M*, assuming no other sources of ecologic bias. The ecologic bias also equals the sum of the individual-level bias due to ignoring groups and the bias caused by aggregation. The latter is measured by *F*, the inflation factor, equal to *M *- 1.

Subtracting (*b*_*c *_- *b*_*w*_) from both sides of equation A15 yields:

(*b*_*e *_- *b*_*c*_) = (*b*_*c *_- *b*_*w*_)(*M *- 1) = (*b*_*c *_- *b*_*w*_) *F*

where *F *= *M *- 1 is called the *inflation factor *[10,16]. Equation A16 measures the amount of bias added by aggregation (Figure 9).

The simplified equation describing bias magnification of confounding by group (equation 10 in the main text) can be derived in another way: insert *y*_*ij *_= *q*_*i *_+ *bx*_*ij *_+ *e*_*ij *_into equation A10 and expand; insert *Y*_*i *_= *q*_*i *_+ *bX*_*i *_+ *e*_*i *_into equation A11 and expand:

(bc−b)=cov⁡[qi,xij]var⁡[xij]
 MathType@MTEF@5@5@+=feaafiart1ev1aaatCvAUfKttLearuWrP9MDH5MBPbIqV92AaeXatLxBI9gBaebbnrfifHhDYfgasaacH8akY=wiFfYdH8Gipec8Eeeu0xXdbba9frFj0=OqFfea0dXdd9vqai=hGuQ8kuc9pgc9s8qqaq=dirpe0xb9q8qiLsFr0=vr0=vr0dc8meaabaqaciaacaGaaeqabaqabeGadaaakeaadaqadaqaaiabdkgaInaaBaaaleaacqWGJbWyaeqaaOGaeyOeI0IaemOyaigacaGLOaGaayzkaaGaeyypa0ZaaSaaaeaacyGGJbWycqGGVbWBcqGG2bGDcqGGBbWwcqWGXbqCdaWgaaWcbaGaemyAaKgabeaakiabcYcaSiabdIha4naaBaaaleaacqWGPbqAcqWGQbGAaeqaaOGaeiyxa0fabaGagiODayNaeiyyaeMaeiOCaiNaei4waSLaemiEaG3aaSbaaSqaaiabdMgaPjabdQgaQbqabaGccqGGDbqxaaaaaa@4E57@

(be−b)=cov⁡B[qi,Xi]var⁡B[Xi]
 MathType@MTEF@5@5@+=feaafiart1ev1aaatCvAUfKttLearuWrP9MDH5MBPbIqV92AaeXatLxBI9gBaebbnrfifHhDYfgasaacH8akY=wiFfYdH8Gipec8Eeeu0xXdbba9frFj0=OqFfea0dXdd9vqai=hGuQ8kuc9pgc9s8qqaq=dirpe0xb9q8qiLsFr0=vr0=vr0dc8meaabaqaciaacaGaaeqabaqabeGadaaakeaadaqadaqaaiabdkgaInaaBaaaleaacqWGLbqzaeqaaOGaeyOeI0IaemOyaigacaGLOaGaayzkaaGaeyypa0ZaaSaaaeaacyGGJbWycqGGVbWBcqGG2bGDdaWgaaWcbaGaeeOqaieabeaakiabcUfaBjabdghaXnaaBaaaleaacqWGPbqAaeqaaOGaeiilaWIaemiwaG1aaSbaaSqaaiabdMgaPbqabaGccqGGDbqxaeaacyGG2bGDcqGGHbqycqGGYbGCdaWgaaWcbaGaeeOqaieabeaakiabcUfaBjabdIfaynaaBaaaleaacqWGPbqAaeqaaOGaeiyxa0faaaaa@4DA3@

Dividing equation A18 by equation A17 yields:

(be−b)(bc−b)=cov⁡B[qi,Xi]cov⁡[qi,xij]var⁡[xij]var⁡B[Xi]=M
 MathType@MTEF@5@5@+=feaafiart1ev1aaatCvAUfKttLearuWrP9MDH5MBPbIqV92AaeXatLxBI9gBaebbnrfifHhDYfgasaacH8akY=wiFfYdH8Gipec8Eeeu0xXdbba9frFj0=OqFfea0dXdd9vqai=hGuQ8kuc9pgc9s8qqaq=dirpe0xb9q8qiLsFr0=vr0=vr0dc8meaabaqaciaacaGaaeqabaqabeGadaaakeaadaWcaaqaamaabmaabaGaemOyai2aaSbaaSqaaiabdwgaLbqabaGccqGHsislcqWGIbGyaiaawIcacaGLPaaaaeaadaqadaqaaiabdkgaInaaBaaaleaacqWGJbWyaeqaaOGaeyOeI0IaemOyaigacaGLOaGaayzkaaaaaiabg2da9maalaaabaGagi4yamMaei4Ba8MaeiODay3aaSbaaSqaaiabbkeacbqabaGccqGGBbWwcqWGXbqCdaWgaaWcbaGaemyAaKgabeaakiabcYcaSiabdIfaynaaBaaaleaacqWGPbqAaeqaaOGaeiyxa0fabaGagi4yamMaei4Ba8MaeiODayNaei4waSLaemyCae3aaSbaaSqaaiabdMgaPbqabaGccqGGSaalcqWG4baEdaWgaaWcbaGaemyAaKMaemOAaOgabeaakiabc2faDbaadaWcaaqaaiGbcAha2jabcggaHjabckhaYjabcUfaBjabdIha4naaBaaaleaacqWGPbqAcqWGQbGAaeqaaOGaeiyxa0fabaGagiODayNaeiyyaeMaeiOCai3aaSbaaSqaaiabbkeacbqabaGccqGGBbWwcqWGybawdaWgaaWcbaGaemyAaKgabeaakiabc2faDbaacqGH9aqpcqWGnbqtaaa@7081@

Note that cov[*q*_*i*_, *x*_*ij*_] = cov_B_[*q*_*i*_, *X*_*i*_], a consequence of using population weighting. Confounding by group is absent if cov_B_[*q*_*i*_, *X*_*i*_] = 0, i.e., the background risks and average exposures are uncorrelated (e.g., *q*_0 _= *q*_1 _for two 2 × 2 tables).

When *M *equals one – i.e., exposure within groups is homogeneous – equation A15 shows that the ecologic and individual-level results are equal. This result, desirable for the nominally ecologic study, is a consequence of using population weighting; unweighted ecologic regression will generally produce different results from the individual-level analysis. For example, in (A19) the weighted and unweighted covariances of *X*_*i *_and *q*_*i *_will usually not be equal [11].

### 4. M ≥ 1 when *X*_*i *_is the ecologic exposure measure

Suppose we use the mean exposure in each group (*X*_*i*_) as our ecologic measure of exposure. Partitioning total exposure variance within and between groups shows that *M *is always at least one:

M=var⁡[xij]var⁡B[Xi]=VTVB=VW+VBVB≥1
 MathType@MTEF@5@5@+=feaafiart1ev1aaatCvAUfKttLearuWrP9MDH5MBPbIqV92AaeXatLxBI9gBaebbnrfifHhDYfgasaacH8akY=wiFfYdH8Gipec8Eeeu0xXdbba9frFj0=OqFfea0dXdd9vqai=hGuQ8kuc9pgc9s8qqaq=dirpe0xb9q8qiLsFr0=vr0=vr0dc8meaabaqaciaacaGaaeqabaqabeGadaaakeaacqWGnbqtcqGH9aqpdaWcaaqaaiGbcAha2jabcggaHjabckhaYjabcUfaBjabdIha4naaBaaaleaacqWGPbqAcqWGQbGAaeqaaOGaeiyxa0fabaGagiODayNaeiyyaeMaeiOCai3aaSbaaSqaaiabbkeacbqabaGccqGGBbWwcqWGybawdaWgaaWcbaGaemyAaKgabeaakiabc2faDbaacqGH9aqpdaWcaaqaaiabdAfawnaaBaaaleaacqWGubavaeqaaaGcbaGaemOvay1aaSbaaSqaaiabdkeacbqabaaaaOGaeyypa0ZaaSaaaeaacqWGwbGvdaWgaaWcbaGaem4vaCfabeaakiabgUcaRiabdAfawnaaBaaaleaacqWGcbGqaeqaaaGcbaGaemOvay1aaSbaaSqaaiabdkeacbqabaaaaOGaeyyzImRaeGymaedaaa@5719@

### 5. Effect measure modification and *b*_*c*_

Using the notation in Table 6, the crude risk difference is

bc=∑ipimi1∑imi1−∑iqimi0∑imi0
 MathType@MTEF@5@5@+=feaafiart1ev1aaatCvAUfKttLearuWrP9MDH5MBPbIqV92AaeXatLxBI9gBaebbnrfifHhDYfgasaacH8akY=wiFfYdH8Gipec8Eeeu0xXdbba9frFj0=OqFfea0dXdd9vqai=hGuQ8kuc9pgc9s8qqaq=dirpe0xb9q8qiLsFr0=vr0=vr0dc8meaabaqaciaacaGaaeqabaqabeGadaaakeaacqWGIbGydaWgaaWcbaGaem4yamgabeaakiabg2da9maalaaabaWaaabuaeaacqWGWbaCdaWgaaWcbaGaemyAaKgabeaakiabd2gaTnaaBaaaleaacqWGPbqAcqaIXaqmaeqaaaqaaiabdMgaPbqab0GaeyyeIuoaaOqaamaaqafabaGaemyBa02aaSbaaSqaaiabdMgaPjabigdaXaqabaaabaGaemyAaKgabeqdcqGHris5aaaakiabgkHiTmaalaaabaWaaabuaeaacqWGXbqCdaWgaaWcbaGaemyAaKgabeaakiabd2gaTnaaBaaaleaacqWGPbqAcqaIWaamaeqaaaqaaiabdMgaPbqab0GaeyyeIuoaaOqaamaaqafabaGaemyBa02aaSbaaSqaaiabdMgaPjabicdaWaqabaaabaGaemyAaKgabeqdcqGHris5aaaaaaa@54D5@

where *p*_*i *_is the risk in the exposed, *q*_*i *_is the risk in the unexposed and we sum over groups *i*.

To consider effect modification of the risk difference, assume *q*_*i *_= *q *and *p*_*i *_= *q *+ *b*_*i*_. Substituting into (A21), we obtain

bc=∑ibimi1∑imi1=∑ibiwi
 MathType@MTEF@5@5@+=feaafiart1ev1aaatCvAUfKttLearuWrP9MDH5MBPbIqV92AaeXatLxBI9gBaebbnrfifHhDYfgasaacH8akY=wiFfYdH8Gipec8Eeeu0xXdbba9frFj0=OqFfea0dXdd9vqai=hGuQ8kuc9pgc9s8qqaq=dirpe0xb9q8qiLsFr0=vr0=vr0dc8meaabaqaciaacaGaaeqabaqabeGadaaakeaacqWGIbGydaWgaaWcbaGaem4yamgabeaakiabg2da9maalaaabaWaaabuaeaacqWGIbGydaWgaaWcbaGaemyAaKgabeaakiabd2gaTnaaBaaaleaacqWGPbqAcqaIXaqmaeqaaaqaaiabdMgaPbqab0GaeyyeIuoaaOqaamaaqafabaGaemyBa02aaSbaaSqaaiabdMgaPjabigdaXaqabaaabaGaemyAaKgabeqdcqGHris5aaaakiabg2da9maaqafabaGaemOyai2aaSbaaSqaaiabdMgaPbqabaGccqWG3bWDdaWgaaWcbaGaemyAaKgabeaaaeaacqWGPbqAaeqaniabggHiLdaaaa@4C75@

where the *w*_*i *_are non-negative weights. Thus *b*_*c *_must be between the minimum and maximum values of *b*_*i*_. For additional discussion of when *b*_*c *_and *b*_*e *_are bounded, see [11].

### 6. Computation of *b*_*w *_for Table 3, Figure 6

*b*_*w *_is a weighted average of the *b*_*i *_using weights equal to group size times the exposure variance within the group:

*w*_0 _= *n*_0 _var_0_(*x*_0*j*_) = 200(*X*_0_)(1-*X*_0_) = 200(0.5)(0.5) = 50

*w*_1 _= *n*_1 _var_1_(*x*_1*j*_) = 200(*X*_1_)(1-*X*_1_) = 200(0.4)(0.6) = 48

bw=w0b0+w1b1w0+w1=50(0.1)+48(0.5)50+48≈0.296
 MathType@MTEF@5@5@+=feaafiart1ev1aaatCvAUfKttLearuWrP9MDH5MBPbIqV92AaeXatLxBI9gBaebbnrfifHhDYfgasaacH8akY=wiFfYdH8Gipec8Eeeu0xXdbba9frFj0=OqFfea0dXdd9vqai=hGuQ8kuc9pgc9s8qqaq=dirpe0xb9q8qiLsFr0=vr0=vr0dc8meaabaqaciaacaGaaeqabaqabeGadaaakqaaeeqaaiabdkgaInaaBaaaleaacqWG3bWDaeqaaOGaeyypa0ZaaSaaaeaacqWG3bWDdaWgaaWcbaGaeGimaadabeaakiabdkgaInaaBaaaleaacqaIWaamaeqaaOGaey4kaSIaem4DaC3aaSbaaSqaaiabigdaXaqabaGccqWGIbGydaWgaaWcbaGaeGymaedabeaaaOqaaiabdEha3naaBaaaleaacqaIWaamaeqaaOGaey4kaSIaem4DaC3aaSbaaSqaaiabigdaXaqabaaaaaGcbaGaeyypa0ZaaSaaaeaacqaI1aqncqaIWaamcqGGOaakcqaIWaamcqGGUaGlcqaIXaqmcqGGPaqkcqGHRaWkcqaI0aancqaI4aaocqGGOaakcqaIWaamcqGGUaGlcqaI1aqncqGGPaqkaeaacqaI1aqncqaIWaamcqGHRaWkcqaI0aancqaI4aaoaaGaeyisISRaeGimaaJaeiOla4IaeGOmaiJaeGyoaKJaeGOnaydaaaa@5BCB@

### 7. Non-differential exposure misclassification

From Table 4, the average exposure in the misclassified two by two table is

Ui=s(ai+ci)+(1−t)(bi+di)ni=s(ai+ci)ni+(1−t)(ni−ai−ci)ni=sXi+(1−t)(1−Xi)=(s+t−1)Xi+(1−t)=λXi+(1−t)
 MathType@MTEF@5@5@+=feaafiart1ev1aaatCvAUfKttLearuWrP9MDH5MBPbIqV92AaeXatLxBI9gBaebbnrfifHhDYfgasaacH8akY=wiFfYdH8Gipec8Eeeu0xXdbba9frFj0=OqFfea0dXdd9vqai=hGuQ8kuc9pgc9s8qqaq=dirpe0xb9q8qiLsFr0=vr0=vr0dc8meaabaqaciaacaGaaeqabaqabeGadaaakqaaeeqaaiabdwfavnaaBaaaleaacqWGPbqAaeqaaOGaeyypa0ZaaSaaaeaacqWGZbWCcqGGOaakcqWGHbqydaWgaaWcbaGaemyAaKgabeaakiabgUcaRiabdogaJnaaBaaaleaacqWGPbqAaeqaaOGaeiykaKIaey4kaSIaeiikaGIaeGymaeJaeyOeI0IaemiDaqNaeiykaKIaeiikaGIaemOyai2aaSbaaSqaaiabdMgaPbqabaGccqGHRaWkcqWGKbazdaWgaaWcbaGaemyAaKgabeaakiabcMcaPaqaaiabd6gaUnaaBaaaleaacqWGPbqAaeqaaaaaaOqaaiabg2da9iabdohaZnaalaaabaGaeiikaGIaemyyae2aaSbaaSqaaiabdMgaPbqabaGccqGHRaWkcqWGJbWydaWgaaWcbaGaemyAaKgabeaakiabcMcaPaqaaiabd6gaUnaaBaaaleaacqWGPbqAaeqaaaaakiabgUcaRiabcIcaOiabigdaXiabgkHiTiabdsha0jabcMcaPmaalaaabaGaeiikaGIaemOBa42aaSbaaSqaaiabdMgaPbqabaGccqGHsislcqWGHbqydaWgaaWcbaGaemyAaKgabeaakiabgkHiTiabdogaJnaaBaaaleaacqWGPbqAaeqaaOGaeiykaKcabaGaemOBa42aaSbaaSqaaiabdMgaPbqabaaaaaGcbaGaeyypa0Jaem4CamNaemiwaG1aaSbaaSqaaiabdMgaPbqabaGccqGHRaWkcqGGOaakcqaIXaqmcqGHsislcqWG0baDcqGGPaqkcqGGOaakcqaIXaqmcqGHsislcqWGybawdaWgaaWcbaGaemyAaKgabeaakiabcMcaPaqaaiabg2da9iabcIcaOiabdohaZjabgUcaRiabdsha0jabgkHiTiabigdaXiabcMcaPiabdIfaynaaBaaaleaacqWGPbqAaeqaaOGaey4kaSIaeiikaGIaeGymaeJaeyOeI0IaemiDaqNaeiykaKcabaGaeyypa0dcciGae83UdWMaemiwaG1aaSbaaSqaaiabdMgaPbqabaGccqGHRaWkcqGGOaakcqaIXaqmcqGHsislcqWG0baDcqGGPaqkaaaa@9C59@

where *λ *= *s*+*t*-1. Assuming *s *and *t *are the same in all groups, the ecologic estimate of the RD for the misclassified data is

be′=cov⁡B[Yi,Ui]var⁡B[Ui]=cov⁡B[Yi,λXi+(1−t)]var⁡B[λXi+(1−t)]=λcov⁡B[Yi,Xi]λ2var⁡B[Xi]=beλ
 MathType@MTEF@5@5@+=feaafiart1ev1aaatCvAUfKttLearuWrP9MDH5MBPbIqV92AaeXatLxBI9gBamXvP5wqSXMqHnxAJn0BKvguHDwzZbqegyvzYrwyUfgarqqtubsr4rNCHbGeaGqiA8vkIkVAFgIELiFeLkFeLk=iY=Hhbbf9v8qqaqFr0xc9pk0xbba9q8WqFfeaY=biLkVcLq=JHqVepeea0=as0db9vqpepesP0xe9Fve9Fve9GapdbaqaaeGacaGaaiaabeqaamqadiabaaGceaabbeaacqWGIbGydaqhaaWcbaGaemyzaugabaGccWaGGBOmGikaaiabg2da9maalaaabaGagi4yamMaei4Ba8MaeiODay3aaSbaaSqaaiabbkeacbqabaGccqGGBbWwcqWGzbqwdaWgaaWcbaGaemyAaKgabeaakiabcYcaSiabdwfavnaaBaaaleaacqWGPbqAaeqaaOGaeiyxa0fabaGagiODayNaeiyyaeMaeiOCai3aaSbaaSqaaiabbkeacbqabaGccqGGBbWwcqWGvbqvdaWgaaWcbaGaemyAaKgabeaakiabc2faDbaaaeaacqGH9aqpdaWcaaqaaiGbcogaJjabc+gaVjabcAha2naaBaaaleaacqqGcbGqaeqaaOGaei4waSLaemywaK1aaSbaaSqaaiabdMgaPbqabaGccqGGSaaliiGacqWF7oaBcqWGybawdaWgaaWcbaGaemyAaKgabeaakiabgUcaRiabcIcaOiabigdaXiabgkHiTiabdsha0jabcMcaPiabc2faDbqaaiGbcAha2jabcggaHjabckhaYnaaBaaaleaacqqGcbGqaeqaaOGaei4waSLae83UdWMaemiwaG1aaSbaaSqaaiabdMgaPbqabaGccqGHRaWkcqGGOaakcqaIXaqmcqGHsislcqWG0baDcqGGPaqkcqGGDbqxaaaabaGaeyypa0ZaaSaaaeaacqWF7oaBcyGGJbWycqGGVbWBcqGG2bGDdaWgaaWcbaGaeeOqaieabeaakiabcUfaBjabdMfaznaaBaaaleaacqWGPbqAaeqaaOGaeiilaWIaemiwaG1aaSbaaSqaaiabdMgaPbqabaGccqGGDbqxaeaacqWF7oaBdaahaaWcbeqaaiabikdaYaaakiGbcAha2jabcggaHjabckhaYnaaBaaaleaacqqGcbGqaeqaaOGaei4waSLaemiwaG1aaSbaaSqaaiabdMgaPbqabaGccqGGDbqxaaaabaGaeyypa0ZaaSaaaeaacqWGIbGydaWgaaWcbaGaemyzaugabeaaaOqaaiab=T7aSbaaaaaa@AA84@

Since 0 ≤ *λ *≤ 1, the variance of *U*_*i *_is smaller than the variance of the *X*_*i*_, i.e., the *U*_*i *_are closer together. If we think of equation A26 as an iteration equation, then NDEM moves the average exposure one step closer to the stationary point given by solving *X*_*c *_= *λX*_*c *_+ (1-*t*):

Xc=1−t1−λ=1−t(1−s)+(1−t)
 MathType@MTEF@5@5@+=feaafiart1ev1aaatCvAUfKttLearuWrP9MDH5MBPbIqV92AaeXatLxBI9gBaebbnrfifHhDYfgasaacH8akY=wiFfYdH8Gipec8Eeeu0xXdbba9frFj0=OqFfea0dXdd9vqai=hGuQ8kuc9pgc9s8qqaq=dirpe0xb9q8qiLsFr0=vr0=vr0dc8meaabaqaciaacaGaaeqabaqabeGadaaakeaacqWGybawdaWgaaWcbaGaem4yamgabeaakiabg2da9maalaaabaGaeGymaeJaeyOeI0IaemiDaqhabaGaeGymaeJaeyOeI0ccciGae83UdWgaaiabg2da9maalaaabaGaeGymaeJaeyOeI0IaemiDaqhabaGaeiikaGIaeGymaeJaeyOeI0Iaem4CamNaeiykaKIaey4kaSIaeiikaGIaeGymaeJaeyOeI0IaemiDaqNaeiykaKcaaaaa@46AA@

For *s *= *t*, *X*_*c *_= 0.5.

In the absence of any other sources of bias except NDEM, one can show that the individual-level estimate of the RD is given by

bcb=λvar⁡[xij]var⁡[uij]≤1
 MathType@MTEF@5@5@+=feaafiart1ev1aaatCvAUfKttLearuWrP9MDH5MBPbIqV92AaeXatLxBI9gBaebbnrfifHhDYfgasaacH8akY=wiFfYdH8Gipec8Eeeu0xXdbba9frFj0=OqFfea0dXdd9vqai=hGuQ8kuc9pgc9s8qqaq=dirpe0xb9q8qiLsFr0=vr0=vr0dc8meaabaqaciaacaGaaeqabaqabeGadaaakeaadaWcaaqaaiabdkgaInaaBaaaleaacqWGJbWyaeqaaaGcbaGaemOyaigaaiabg2da9GGaciab=T7aSnaalaaabaGagiODayNaeiyyaeMaeiOCaiNaei4waSLaemiEaG3aaSbaaSqaaiabdMgaPjabdQgaQbqabaGccqGGDbqxaeaacyGG2bGDcqGGHbqycqGGYbGCcqGGBbWwcqWG1bqDdaWgaaWcbaGaemyAaKMaemOAaOgabeaakiabc2faDbaacqGHKjYOcqaIXaqmaaa@4C71@

where *u*_*ij *_is the misclassified exposure on the individual level (If we allow values of *s *and *t *below 0.5, the absolute value of (A29) is less than or equal to unity). Using (A14), we can expand (A29) into within-group (left term) and between-group (right term) portions:

(bcb)=(λvar⁡W[xij]var⁡W[uij])(var⁡W[uij]var⁡[uij])+(1λ)(1MU)
 MathType@MTEF@5@5@+=feaafiart1ev1aaatCvAUfKttLearuWrP9MDH5MBPbIqV92AaeXatLxBI9gBaebbnrfifHhDYfgasaacH8akY=wiFfYdH8Gipec8Eeeu0xXdbba9frFj0=OqFfea0dXdd9vqai=hGuQ8kuc9pgc9s8qqaq=dirpe0xb9q8qiLsFr0=vr0=vr0dc8meaabaqaciaacaGaaeqabaqabeGadaaakeaadaqadaqaamaalaaabaGaemOyai2aaSbaaSqaaiabdogaJbqabaaakeaacqWGIbGyaaaacaGLOaGaayzkaaGaeyypa0ZaaeWaaeaaiiGacqWF7oaBdaWcaaqaaiGbcAha2jabcggaHjabckhaYnaaBaaaleaacqqGxbWvaeqaaOGaei4waSLaemiEaG3aaSbaaSqaaiabdMgaPjabdQgaQbqabaGccqGGDbqxaeaacyGG2bGDcqGGHbqycqGGYbGCdaWgaaWcbaGaee4vaCfabeaakiabcUfaBjabdwha1naaBaaaleaacqWGPbqAcqWGQbGAaeqaaOGaeiyxa0faaaGaayjkaiaawMcaamaabmaabaWaaSaaaeaacyGG2bGDcqGGHbqycqGGYbGCdaWgaaWcbaGaee4vaCfabeaakiabcUfaBjabdwha1naaBaaaleaacqWGPbqAcqWGQbGAaeqaaOGaeiyxa0fabaGagiODayNaeiyyaeMaeiOCaiNaei4waSLaemyDau3aaSbaaSqaaiabdMgaPjabdQgaQbqabaGccqGGDbqxaaaacaGLOaGaayzkaaGaey4kaSYaaeWaaeaadaWcaaqaaiabigdaXaqaaiab=T7aSbaaaiaawIcacaGLPaaadaqadaqaamaalaaabaGaeGymaedabaGaemyta00aaSbaaSqaaiabdwfavbqabaaaaaGccaGLOaGaayzkaaaaaa@7301@

where *M*_*U *_is the magnification factor for the misclassified exposure. All expressions in parentheses in equation A30 are less than or equal to one except (1/*λ*). Thus, while the ecologic estimate of the RD is biased away from the null by 1/*λ*, this tendency is counterbalanced in individual level studies by the inverse of the magnification factor *M*_*U*_. *λM*_*U *_≥ *M *≥ 1, as can be derived from (A29):

bcb=λvar⁡[xij]var⁡[uij]var⁡B[Ui]var⁡B[Ui]var⁡B[Xi]var⁡B[Xi]=λvar⁡[xij]var⁡B[Xi]var⁡B[Ui]var⁡[uij]var⁡B[Xi]var⁡B[Ui]=λM1MUvar⁡B[Xi]λ2var⁡B[Xi]=MλMU≤1
 MathType@MTEF@5@5@+=feaafiart1ev1aaatCvAUfKttLearuWrP9MDH5MBPbIqV92AaeXatLxBI9gBaebbnrfifHhDYfgasaacH8akY=wiFfYdH8Gipec8Eeeu0xXdbba9frFj0=OqFfea0dXdd9vqai=hGuQ8kuc9pgc9s8qqaq=dirpe0xb9q8qiLsFr0=vr0=vr0dc8meaabaqaciaacaGaaeqabaqabeGadaaakqaaeeqaamaalaaabaGaemOyai2aaSbaaSqaaiabdogaJbqabaaakeaacqWGIbGyaaGaeyypa0dcciGae83UdW2aaSaaaeaacyGG2bGDcqGGHbqycqGGYbGCcqGGBbWwcqWG4baEdaWgaaWcbaGaemyAaKMaemOAaOgabeaakiabc2faDbqaaiGbcAha2jabcggaHjabckhaYjabcUfaBjabdwha1naaBaaaleaacqWGPbqAcqWGQbGAaeqaaOGaeiyxa0faamaalaaabaGagiODayNaeiyyaeMaeiOCai3aaSbaaSqaaiabbkeacbqabaGccqGGBbWwcqWGvbqvdaWgaaWcbaGaemyAaKgabeaakiabc2faDbqaaiGbcAha2jabcggaHjabckhaYnaaBaaaleaacqqGcbGqaeqaaOGaei4waSLaemyvau1aaSbaaSqaaiabdMgaPbqabaGccqGGDbqxaaWaaSaaaeaacyGG2bGDcqGGHbqycqGGYbGCdaWgaaWcbaGaeeOqaieabeaakiabcUfaBjabdIfaynaaBaaaleaacqWGPbqAaeqaaOGaeiyxa0fabaGagiODayNaeiyyaeMaeiOCai3aaSbaaSqaaiabbkeacbqabaGccqGGBbWwcqWGybawdaWgaaWcbaGaemyAaKgabeaakiabc2faDbaaaeaacqGH9aqpcqWF7oaBdaWcaaqaaiGbcAha2jabcggaHjabckhaYjabcUfaBjabdIha4naaBaaaleaacqWGPbqAcqWGQbGAaeqaaOGaeiyxa0fabaGagiODayNaeiyyaeMaeiOCai3aaSbaaSqaaiabbkeacbqabaGccqGGBbWwcqWGybawdaWgaaWcbaGaemyAaKgabeaakiabc2faDbaadaWcaaqaaiGbcAha2jabcggaHjabckhaYnaaBaaaleaacqqGcbGqaeqaaOGaei4waSLaemyvau1aaSbaaSqaaiabdMgaPbqabaGccqGGDbqxaeaacyGG2bGDcqGGHbqycqGGYbGCcqGGBbWwcqWG1bqDdaWgaaWcbaGaemyAaKMaemOAaOgabeaakiabc2faDbaadaWcaaqaaiGbcAha2jabcggaHjabckhaYnaaBaaaleaacqqGcbGqaeqaaOGaei4waSLaemiwaG1aaSbaaSqaaiabdMgaPbqabaGccqGGDbqxaeaacyGG2bGDcqGGHbqycqGGYbGCdaWgaaWcbaGaeeOqaieabeaakiabcUfaBjabdwfavnaaBaaaleaacqWGPbqAaeqaaOGaeiyxa0faaaqaaiabg2da9iab=T7aSjabd2eannaalaaabaGaeGymaedabaGaemyta00aaSbaaSqaaiabdwfavbqabaaaaOWaaSaaaeaacyGG2bGDcqGGHbqycqGGYbGCdaWgaaWcbaGaeeOqaieabeaakiabcUfaBjabdIfaynaaBaaaleaacqWGPbqAaeqaaOGaeiyxa0fabaGae83UdW2aaWbaaSqabeaacqaIYaGmaaGccyGG2bGDcqGGHbqycqGGYbGCdaWgaaWcbaGaeeOqaieabeaakiabcUfaBjabdIfaynaaBaaaleaacqWGPbqAaeqaaOGaeiyxa0faaaqaaiabg2da9maalaaabaGaemyta0eabaGae83UdWMaemyta00aaSbaaSqaaiabdwfavbqabaaaaOGaeyizImQaeGymaedaaaa@E170@

### 8. Computation of *M *when exposure is binary

When exposure *x*_*ij *_is binary, *M *can be computed from ecologic exposure data (*X*_*i*_) alone. Calculate the total individual-level exposure variance using the standard equation:

var⁡[xij]=X¯(1−X¯)
 MathType@MTEF@5@5@+=feaafiart1ev1aaatCvAUfKttLearuWrP9MDH5MBPbIqV92AaeXatLxBI9gBaebbnrfifHhDYfgasaacH8akY=wiFfYdH8Gipec8Eeeu0xXdbba9frFj0=OqFfea0dXdd9vqai=hGuQ8kuc9pgc9s8qqaq=dirpe0xb9q8qiLsFr0=vr0=vr0dc8meaabaqaciaacaGaaeqabaqabeGadaaakeaacyGG2bGDcqGGHbqycqGGYbGCcqGGBbWwcqWG4baEdaWgaaWcbaGaemyAaKMaemOAaOgabeaakiabc2faDjabg2da9iqbdIfayzaaraGaeiikaGIaeGymaeJaeyOeI0IafmiwaGLbaebacqGGPaqkaaa@3EF6@

where X¯
 MathType@MTEF@5@5@+=feaafiart1ev1aaatCvAUfKttLearuWrP9MDH5MBPbIqV92AaeXatLxBI9gBaebbnrfifHhDYfgasaacH8akY=wiFfYdH8Gipec8Eeeu0xXdbba9frFj0=OqFfea0dXdd9vqai=hGuQ8kuc9pgc9s8qqaq=dirpe0xb9q8qiLsFr0=vr0=vr0dc8meaabaqaciaacaGaaeqabaqabeGadaaakeaacuWGybawgaqeaaaa@2DFD@ is the population-weighted mean of the *X*_*i*_. Use equation A13 for var_B_[*X*_*i*_].

### 9. Confounding within groups

Assume a reference individual-level model with a linear relationship between outcome and exposure. Assume that outcome is also related to an individual-level covariate *z*_*ij *_via a possibly nonlinear function *h*():

*y*_*ij *_= *q *+ *bx*_*ij *_+ *h*(*z*_*ij*_) + *e*_*ij*_

Aggregation produces

*Y*_*i *_= *q *+ *bX*_*i *_+ *H*_*i*_(*z*_*ij*_) + *e*_*i*_

Hi(zij)=1ni∑j=1nih(zij)
 MathType@MTEF@5@5@+=feaafiart1ev1aaatCvAUfKttLearuWrP9MDH5MBPbIqV92AaeXatLxBI9gBaebbnrfifHhDYfgasaacH8akY=wiFfYdH8Gipec8Eeeu0xXdbba9frFj0=OqFfea0dXdd9vqai=hGuQ8kuc9pgc9s8qqaq=dirpe0xb9q8qiLsFr0=vr0=vr0dc8meaabaqaciaacaGaaeqabaqabeGadaaakeaacqWGibasdaWgaaWcbaGaemyAaKgabeaakiabcIcaOiabdQha6naaBaaaleaacqWGPbqAcqWGQbGAaeqaaOGaeiykaKIaeyypa0ZaaSaaaeaacqaIXaqmaeaacqWGUbGBdaWgaaWcbaGaemyAaKgabeaaaaGcdaaeWbqaaiabdIgaOjabcIcaOiabdQha6naaBaaaleaacqWGPbqAcqWGQbGAaeqaaOGaeiykaKcaleaacqWGQbGAcqGH9aqpcqaIXaqmaeaacqWGUbGBdaWgaaadbaGaemyAaKgabeaaa0GaeyyeIuoaaaa@4A67@

where *H*_*i*_(*z*_*ij*_) is the average value of *h*(*z*_*ij*_) within group *i*. The crude individual-level estimate *b*_*c *_is derived by inserting equation A33 into equation A10 and expanding:

bc=b+cov⁡[xij,h(zij)]var⁡[xij]
 MathType@MTEF@5@5@+=feaafiart1ev1aaatCvAUfKttLearuWrP9MDH5MBPbIqV92AaeXatLxBI9gBaebbnrfifHhDYfgasaacH8akY=wiFfYdH8Gipec8Eeeu0xXdbba9frFj0=OqFfea0dXdd9vqai=hGuQ8kuc9pgc9s8qqaq=dirpe0xb9q8qiLsFr0=vr0=vr0dc8meaabaqaciaacaGaaeqabaqabeGadaaakeaacqWGIbGydaWgaaWcbaGaem4yamgabeaakiabg2da9iabdkgaIjabgUcaRmaalaaabaGagi4yamMaei4Ba8MaeiODayNaei4waSLaemiEaG3aaSbaaSqaaiabdMgaPjabdQgaQbqabaGccqGGSaalcqWGObaAcqGGOaakcqWG6bGEdaWgaaWcbaGaemyAaKMaemOAaOgabeaakiabcMcaPiabc2faDbqaaiGbcAha2jabcggaHjabckhaYjabcUfaBjabdIha4naaBaaaleaacqWGPbqAcqWGQbGAaeqaaOGaeiyxa0faaaaa@513D@

Partitioning the covariance within and between groups yields

bc=b+cov⁡W[xij,h(zij)]var⁡[xij]+cov⁡B[Xi,Hi(zij)]var⁡[xij]
 MathType@MTEF@5@5@+=feaafiart1ev1aaatCvAUfKttLearuWrP9MDH5MBPbIqV92AaeXatLxBI9gBaebbnrfifHhDYfgasaacH8akY=wiFfYdH8Gipec8Eeeu0xXdbba9frFj0=OqFfea0dXdd9vqai=hGuQ8kuc9pgc9s8qqaq=dirpe0xb9q8qiLsFr0=vr0=vr0dc8meaabaqaciaacaGaaeqabaqabeGadaaakeaacqWGIbGydaWgaaWcbaGaem4yamgabeaakiabg2da9iabdkgaIjabgUcaRmaalaaabaGagi4yamMaei4Ba8MaeiODay3aaSbaaSqaaiabcEfaxbqabaGccqGGBbWwcqWG4baEdaWgaaWcbaGaemyAaKMaemOAaOgabeaakiabcYcaSiabdIgaOjabcIcaOiabdQha6naaBaaaleaacqWGPbqAcqWGQbGAaeqaaOGaeiykaKIaeiyxa0fabaGagiODayNaeiyyaeMaeiOCaiNaei4waSLaemiEaG3aaSbaaSqaaiabdMgaPjabdQgaQbqabaGccqGGDbqxaaGaey4kaSYaaSaaaeaacyGGJbWycqGGVbWBcqGG2bGDdaWgaaWcbaGaeeOqaieabeaakiabcUfaBjabdIfaynaaBaaaleaacqWGPbqAaeqaaOGaeiilaWIaemisaG0aaSbaaSqaaiabdMgaPbqabaGccqGGOaakcqWG6bGEdaWgaaWcbaGaemyAaKMaemOAaOgabeaakiabcMcaPiabc2faDbqaaiGbcAha2jabcggaHjabckhaYjabcUfaBjabdIha4naaBaaaleaacqWGPbqAcqWGQbGAaeqaaOGaeiyxa0faaaaa@730A@

The ecologic estimate *b*_*e *_is derived by inserting equation A34 into equation A11 and expanding:

be=b+cov⁡B[Xi,Hi(zij)]var⁡B[Xi]
 MathType@MTEF@5@5@+=feaafiart1ev1aaatCvAUfKttLearuWrP9MDH5MBPbIqV92AaeXatLxBI9gBaebbnrfifHhDYfgasaacH8akY=wiFfYdH8Gipec8Eeeu0xXdbba9frFj0=OqFfea0dXdd9vqai=hGuQ8kuc9pgc9s8qqaq=dirpe0xb9q8qiLsFr0=vr0=vr0dc8meaabaqaciaacaGaaeqabaqabeGadaaakeaacqWGIbGydaWgaaWcbaGaemyzaugabeaakiabg2da9iabdkgaIjabgUcaRmaalaaabaGagi4yamMaei4Ba8MaeiODay3aaSbaaSqaaiabbkeacbqabaGccqGGBbWwcqWGybawdaWgaaWcbaGaemyAaKgabeaakiabcYcaSiabdIeainaaBaaaleaacqWGPbqAaeqaaOGaeiikaGIaemOEaO3aaSbaaSqaaiabdMgaPjabdQgaQbqabaGccqGGPaqkcqGGDbqxaeaacyGG2bGDcqGGHbqycqGGYbGCdaWgaaWcbaGaeeOqaieabeaakiabcUfaBjabdIfaynaaBaaaleaacqWGPbqAaeqaaOGaeiyxa0faaaaa@51DA@

The individual-level estimate, equation A37, is biased by two terms: confounding within groups and confounding between groups. The ecologic estimate, equation A38, is biased only by confounding between groups (These results remain true if *h*() is linear, e.g., *h*(*z*_*ij*_) = *γz*_*ij *_and *H*(*z*_*ij*_) = *γZ*_*i*_). Note that an individual-level variable (*z*_*ij*_) can cause confounding between groups even if it doesn't cause confounding within groups. For models like equation A33, confounding within groups alone does not bias the ecologic estimate. For models that are nonlinear in both exposure and covariates, confounding within groups remains important. For further discussion of confounding within groups, see [11,21].

The approach of assuming an individual-level model, aggregating to obtain an ecologic model, and then comparing biases on the individual and group level is very powerful [11].

## Abbreviations

NDEM, non-differential exposure misclassification

OLS, ordinary least squares

RD, risk difference

## Competing interests

The author(s) declare that they have no competing interests.

## Authors' contributions

TW is the sole author of this paper.
